# What Works in Community-Based Interventions Promoting Physical Activity and Healthy Eating? A Review of Reviews

**DOI:** 10.3390/ijerph110605866

**Published:** 2014-05-30

**Authors:** Tilman Brand, Claudia R. Pischke, Berit Steenbock, Johanna Schoenbach, Saskia Poettgen, Florence Samkange-Zeeb, Hajo Zeeb

**Affiliations:** 1 Leibniz Institute for Prevention Research and Epidemiology—BIPS, Achterstrasse 30, 28359 Bremen, Germany; E-Mails: pischke@bips.uni-bremen.de (C.R.P); steenbock@bips.uni-bremen.de (B.S.); schoenbach@bips.uni-bremen.de (J.S.); poettgen@bips.uni-bremen.de (S.P.); samkange@bips.uni-bremen.de (F.S.-Z.); zeeb@bips.uni-bremen.de (H.Z.); 2 Research Focus Health Sciences Bremen, University of Bremen, 28359 Bremen, Germany

**Keywords:** prevention, chronic diseases, health promotion, physical activity, healthy eating, community

## Abstract

Chronic diseases, such as type II diabetes, are on the rise worldwide. There is consistent evidence that physical activity and healthy eating are important lifestyle factors which affect the risk for chronic diseases. Community-based interventions are of particular public health interest as they reach target groups in their natural living environment and may thus achieve high population-level impacts. We conducted a systematic literature search to assess the effectiveness of community-based interventions to promote physical activity and healthy eating. Specifically, we searched for promising intervention strategies in this setting. We narratively summarized the results of 18 systematic reviews. Among children and adolescents, we found moderate evidence for effects on weight change in primary school-aged children for interventions containing a school component. The evidence for interventions aimed at general adult populations was inconclusive. Self-monitoring, group-based components, and motivational signs to encourage stair use were identified as promising strategies to increase physical activity. Among adults at risk for type II diabetes, evidence was found for beneficial effects on weight change and diabetes incidence. However, interventions for this group were not integrated in more comprehensive community-based approaches.

## 1. Introduction

Chronic diseases such as type II diabetes are on the rise worldwide [1]. According to the International Diabetes Federation, 382 million people are currently affected by the disease with an expected increase to 592 million cases globally by the year 2035 [2]. Diabetes and other chronic diseases are closely interlinked. Uncontrolled or undetected type II diabetes contributes to an elevated risk for cardiovascular diseases and can lead to complications associated with conditions with considerable direct and indirect medical costs [3,4]. 

It is known that chronic diseases such as type II diabetes and the resulting complications are largely preventable as they are determined by lifestyle factors, such as diet and physical activity (PA). Being overweight or obese, the consumption of high-fat, high-sugar, low carbohydrate, low fiber diets as well as physical inactivity are widely recognized as key contributors to an increased cardiovascular and diabetes risk [5]. Furthermore, associations with increased all-cause mortality have been established [6,7]. For example, the consumption of low-carbohydrate high-protein diets was associated with higher all-cause mortality in a prospective study examining 85,168 initially disease-free men and women over a period of more than 20 years in the U.S. [6]. Similar increases in all-cause mortality due to a prolonged consumption of diets low in carbohydrate and high in protein have been observed in a large population cohort in Europe [8]. Further evidence at the population-level suggests that a lack of moderate-to-vigorous PA or prolonged sitting time is associated with all-cause mortality [7,9,10], and with elevated risk factors for type II diabetes, such as obesity [11]. Conversely, moderate PA such as walking and the consumption of complex-carbohydrate and low-fat diets are inversely associated with clinical disease endpoints and mortality at the population-level [6,12,13]. 

There is already a large and growing number of studies investigating the effectiveness of community-based interventions to promote PA and healthy eating and several reviews have been conducted to summarize intervention effects for various outcomes and populations [14,15]. Community-based interventions are of particular interest as they reach target groups in their natural living environment and have the potential to achieve high population level impacts [16]. Communities can be defined as geographic areas (e.g., neighborhoods, villages, cities or regions), or as social groups which share a common culture or identity. Both definitions do not exclude one another as in most cases the members of the social groups interact at certain places (e.g., churches). Different definitions of community-based interventions exist [17,18]. Typically, community-based approaches to health promotion and disease prevention emphasize that the individual’s behavior is shaped by a dynamic interplay with the social environment including interpersonal, organizational, cultural, socioeconomic, environmental and policy influences [19,20]. However, interventions differ with regard to the degree to which they address these different levels [21]. Drawing on the typology of McLeroy and colleagues, we distinguish three types of community-based approaches: (1) Communities as a setting/community recruitment: communities are the place where the participants are recruited, but the interventions strategies are mainly individual-focused (e.g., local mass media campaigns, individual counseling); (2) Multi-player or multi-level interventions: the interventions include several components addressing multiple social-ecological levels or multiple stakeholders (working with non-government organizations, e.g., sports clubs, working in several settings, e.g., workplace, shopping malls, community centers); (3) Environmental change interventions: intervention targeting to change the social or physical environment in the community (local policies, availability of recreational facilities for PA, availability of healthy food) [21]. 

Due to the great heterogeneity of community-based approaches, differences in study designs employed in various studies and populations targeted by these interventions, results of existing reviews are still inconclusive. The aim of this paper is to give an up-to-date summary of the current evidence and to analyze intervention effects according to the different intervention strategies and components that were employed as well as the different populations targeted. The information generated in this review of reviews will inform funding bodies, policy makers and service providers about promising strategies to modify physical activity and diet in various population groups.

## 2. Methods

We searched the following databases for systematic reviews and meta-analyses of primary studies on community-based interventions to promote physical activity and healthy eating published: Cochrane Library, PubMed, Campbell Collaboration, Database of Abstracts of Reviews of Effects (DARE, via NICE). In addition, we searched the database of the National Institute for Health and Clinical Excellence (NICE) for evidence summaries. Various combinations of the keywords “prevention”, “promot*”, “intervention”, “physical activity”, “physical inactivity”, “motor activity”, “exercise”, “ergonomic”, “musculoskeletal disorder”, “fitness, “sedentary behave*”, “healthy eating”, “nutrition”, “dietary”, “overweight”, “obese”, “obesity”, “weight”, “body mass index”, “fruit”, “vegetable”, “community”, “neighborhood”, “quarter”, “population-based”, “multi-level”, “multi-component”, “environmental intervention”, “social environment” and “built environment” were used to search for relevant literature (* indicates truncations). The keywords were combined using the Boolean operations OR and AND.

A more detailed search protocol is available as online supplementary material.

Reference lists of potentially relevant systematic reviews of reviews were also perused for reviews that fitted the predefined inclusion criteria. The literature search was conducted by BS in April 2014. Systematic reviews and meta-analyses were included if they fulfilled the following criteria:

- assessed the effectiveness of community-based primary prevention interventions to promote physical activity and healthy eating at the population-level or in at-risk groups (e.g., prediabetes, overweight, inactive individuals)
- presented at least a subgroup analyses for community-based interventions
- published between 2007 and 2014
- included randomized controlled trials and/or other primary studies with non-random control groups and/or other quasi-experimental designs (e.g., time series approaches)
- published in English
- included at least one primary study which was conducted in Europe or America

During the literature search, we focused on reviews that reported to have included community-based intervention studies regardless of reviewer’s definition of the term “community-based” (in most cases no definition was provided). If studies from other specific settings were included in the reviews (e.g., non-public or semi-public areas, such as worksites, schools, health care), we included them if the majority of the primary studies (>80%) were classified as community-based or if the review provided a subsection of a subgroup analysis for the community based studies. We excluded reviews that focused on specific settings such as childcare facilities, schools, occupational or clinical settings because these institutions are not public or semi-public areas and also because there is a distinct body of research for each of these settings. We furthermore excluded reviews focusing on breastfeeding, obesity treatment, malnutrition, cancer prevention, and mental wellbeing, as well as those focusing on very specific groups (e.g., pregnant women, preterm infants, and frail community-dwelling older adults). 

Two authors (BS, JS) selected relevant reviews from the identified full text publications and independently assessed the quality of all selected reviews according to the AMSTAR criteria, an 11-item questionnaire developed to assess the methodological quality of systematic reviews [22,23]. They compared their quality assessment results, discussed the differences and consulted TB where they could not reach consensus. Studies were excluded from this review if they scored ≤4 on the AMSTAR checklist. The results of the AMSTAR rating are available as an online supplementary table (Table S1).

Berit Steenbock, Johanna Schoenbach, Saskia Poettgen and Tilman Brand extracted the study details and core results from all selected reviews and narratively summarized them. As there are varying definitions of the term “community-based”, respectively, no clear definition of the term, we classified the underlying studies of the selected reviews according to the typology defined above: (1) community recruitment, (2) multi-player/multi-level intervention, (3) environmental change interventions. As the AMSTAR criteria mainly focus on the methodological quality of the review and not so much on the evidence of the underlying studies, we briefly appraised the evidence of the reviews we included using the following criteria: adequate sample size in the underlying studies, inclusion of randomized trials, use of objective or validated outcome measures, and inclusion of community-based interventions type 2 or 3. Concerning the question “What works?”, we considered an intervention strategy to be “promising” if it was said to be an effective strategy in at least one systematic review offering at least moderate evidence. 

## 3. Results and Discussion

**Figure 1:**
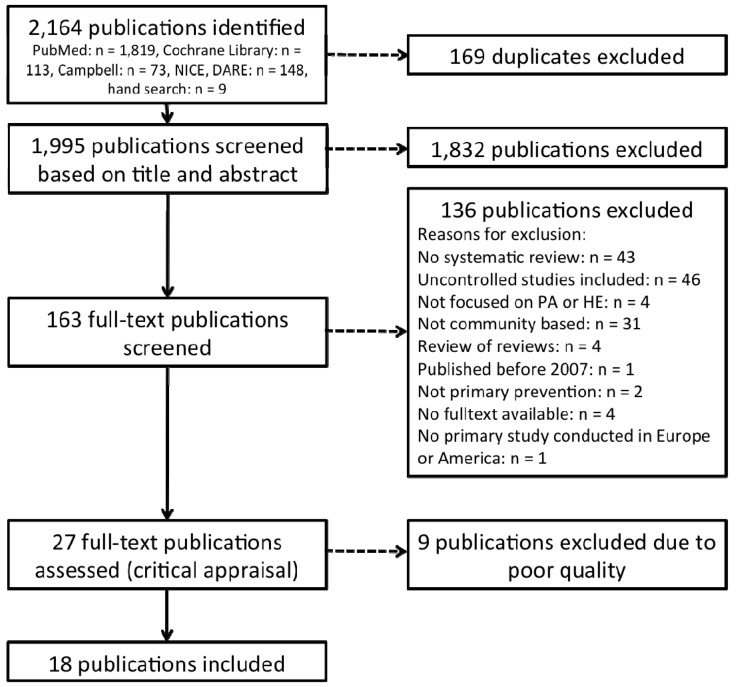
Identification of relevant studies.

The systematic literature search identified 2,164 publications, 163 of which were assessed in detail. Of these, 27 met the selection criteria and were included in the quality assessment. Nine publications were excluded due to poor quality, leaving 18 publications for this review of reviews (Figure 1). The selected publications include nine meta-analyses and nine narrative systematic reviews. Most of the publications were not restricted to community-based interventions but also contained primary studies from other settings (schools, workplaces, health care settings), which we did not take into account for our review. After exclusion of duplications, the selected publications summarize the results of 195 primary studies on community-based interventions. A complete list of the primary studies is available as an online supplement. 

We present the results of our review of reviews separately for the population groups, children and adolescents, adult general population, and at-risk adult population. For each population group we also present the results separately for healthy eating and physical activity.

### 3.1. Children and Adolescents

Seven reviews (five narrative reviews [24,25,26,27,28] and two meta-analyses [29,30]) investigated the effects of community-based interventions to promote healthy eating and physical activity among children and adolescents. Most of the underlying studies focused on children aged 8 to 12 years. None of the reviews included only healthy eating interventions or only PA intervention. Two meta-analyses and two narrative systematic reviews investigated the effects of healthy eating and PA interventions on weight change. Four of the reviews [25,27,28,29] provided only limited evidence due to a small number of included community-based studies, small sample sizes in the underlying studies, and very few multi-level or environmental change interventions. Underlying studies included in the remaining three reviews [24,26,30] included more of the latter interventions and provided moderate evidence (Table 1).

**Table 1:** Children and adolescents: healthy eating and physical activity.

Author	Type of Review/Type of Interventions Included	Sample/Target Group	Intervention Components	Outcome Measures	Main Results	Evidence and Conclusion
Bleich *et al.* 2013 [24]	Narrative systematic review Not community-based: n = 0 Community recruitment: n = 3 Multi-level intervention: n = 2 Environmental change: n = 4	Age: 0–17 years, most studies 8–14 years, 2 studies included only girls, 1 study only boys, sample size: 46–43,811	Community awareness campaigns, group counselling, guided resistance training, dance classes, school-based physical education enhancement, changes in food environment at school, schoolyard garden programs, community capacity building	**HE**: FFQ, direct observation **PA**: Accelerometers, direct observation, self-report **Weight**: BMI/zBMI, fat mass, obesity prevalence	**HE**: no significant effects on consumption of FV, fatty food or sugar sweetened beverage or total energy intake **PA**: significant increase in PA energy expenditure in one study **Weight**: beneficial effects on BMI/zBMI in 4 of the 9 studies.	Moderate evidence: studies with large sample sizes and anthropometric outcome measures included; relatively few studies that were solely community-based; moderate evidence for community-based intervention with a school component, insufficient evidence without school component
Hendrie *et al.* 2011 [25]	Narrative systematic review Not community-based: n = 11 Community recruitment n = 4 Multi-level intervention: n = 0 Environmental change: n = 0	Girls, 8–12 years, in one study both boys and girls included, sample size: 35–61	Summer camp for girls (4 weeks), dance classes, information material, lessons on healthy eating, home work	**HE**: 24 h dietary recall, FV consumption **PA**: Accelerometers (3 studies) and self-report **Weight**: BMI, WC, % body fat	**HE**: no significant effects **PA**: no significant effects **Weight**: no significant effects	Limited evidence: all included studies were pilot studies with small sample sizes; no conclusion can be drawn
Kellou *et al.* 2014 [26]	Narrative systematic review Not community-based: n = 49 Community recruitment: n = 0 Multi-level intervention: n = 2 Environmental change: n = 3	1 study: 0–5 years, 1 study 6–12 years, 3 studies > 13 years, both genders, mostly low SES communities, sample size: 1,001–43,811	Social marketing, school audits, food handlers training, distribution of canteen guidelines, changes in food environment at school, schoolyard garden programs, world food day celebrations, capacity building among school project officers and student ambassadors	**HE**: not assessed in this review **PA**: Accelerometers, direct observation, self-report **Weight**: BMI/zBMI, fat mass, obesity prevalence	**HE**: not assessed in this review **PA**: significant increase in PA energy expenditure in only one study **Weight**: All community-based studies report beneficial effects on either, BMI/zBMI or fat mass	Moderate evidence: studies with large sample sizes and valid measures included, but only non-random control groups, relatively few community-based interventions. Authors view comprehensive approach as most successful
van Sluijs *et al.* 2007 [27]	Narrative systematic review Not community-based: n = 49 Community recruitment: n = 4 Multi-level intervention: n = 0 Environmental change: n = 0	Girls (3 studies), only boys (1 study), 8–14 years, sample size: 35–473	Group activities (“troop meetings”), summer camps, group goal setting, internet based program (also for parents)	**HE**: 24 h dietary recall, FV consumption **PA**: Accelerometers (3 studies) and self-report **Weight**: BMI, WC, % body fat **Other**: bone mineral content and density	**HE**: no significant effects **PA**: no significant effects **Weight**: no significant effects **Bone health**: no significant effects	Limited evidence: small number of community interventions included, mostly pilot studies; no conclusion can be drawn
van Sluijs *et al*. 2011 [28] (updated review)	Narrative systematic review Not community-based: n = 6 Community recruitment: n = 3 Multi-level intervention: n = 0 Environmental change: n = 1	Both genders, 5–16 years, mostly from low SES neighborhoods; sample size: 75–276	School playground made available outside of school hours, variety of equipment provided, computer-tailored storybook, newsletter, curriculum delivered by troop leaders, troop meeting policies, badge assignments, mentoring schemes	**HE**: Youth adolescent FFQ, direct observation, parent and child reports **PA**: Accelerometers (2 studies), direct observation, self-report **Weight**: BMI, WC, fat (free) mass	**HE**: significant reduction in calories in a subgroup of obese children in 1 study **PA**: more children engage in outdoor PA in 1 study based on observation **Weight**: significant decrease in proportion of obese children in 1 study	Limited evidence: small number of studies with community interventions included, effects on HE and PA are restricted to subgroups or direct observation; no conclusion can be drawn
Waters *et al.* 2011 [29]	Meta-analysis Not community-based: n = 48 Community recruitment: n = 5 Multi-level intervention: n = 2 Environmental change: n = 0	Four studies included only girls, the others boys and girls, 4–12 years; sample size: 35–1,235	Advertising media campaigns, Summer camps, dance classes, interactive group sessions (also with parents), individual goal setting, support from dietitians, changes in the school curriculum	**HE**: not assessed in the meta-analysis **PA**: not assessed in the meta-analysis **Weight**: Standardized Mean Difference (SMD) in BMI/zBMI	Effects on BMI/zBMI assessed for “education plus other” (SMD = −0.09, 95% CI: −0.20, 0.02, I^2^ = 56%) and “non educational setting” (SMD = −0.28, 95% CI: −0.72, 0.16, I^2^ = 87%), both included community-based and other studies	Limited evidence: mixture of community-based and other studies, high degree of heterogeneity, re-calculation of effect sizes from some primary studies questionable; Authors find strongest evidence for primary school-aged children
Wolfenden *et al.* 2014 [30]	Meta-analysis Not community-based: n = 0 Community recruitment: n = 1 Multi-level intervention: n = 3 Environmental change: n = 4	1 study: 0–5 years, 3 studies 5–11 years, 4 studies 12–18 years, both genders, sample size: 730–43,811	Social marketing, school audits, food handlers training, distribution of canteen guidelines, changes in food environment at school, schoolyard garden programs, community vegetable garden, removal of soft drink from vending machines, world food day celebrations, capacity building among school project officers and student ambassadors, consultation with health department	**HE**: not assessed in the meta-analysis **PA**: not assessed in the meta-analysis **Weight**: zBMI; % body fat	Combined MD in zBMI = −0.09 (95% CI: −0.16, −0.02, I^2^ = 93%) Subgroup analysis for age groups: MD in zBMI for adolescents (12–18 years) = −0.02 (95% CI: −0.08, 0.03, I^2^ = 70%); primary school-aged children (5–11 years): MD = −0.16 (95% CI: −0.27, −0.05, I^2^ = 92%); preschool children (0–5 years): only 1 study	Moderate evidence: studies with large sample sizes and valid measures included, but high degree of heterogeneity and only non-random control groups. Strongest evidence for primary school-aged children

Notes: BMI: body mass index, CI: confidence interval, FV: fruit and vegetable, HE: healthy eating, MD: mean difference, PA: physical activity, SMD: standardized mean difference, WC: waist circumference, zBMI age and sex-standardized body mass index.

None of the reviews reported significant effects of community-based interventions on healthy eating. In addition, only a few of the underlying studies indicate some intervention effects on PA. With regard to weight change, Waters and colleagues report small, non-significant effects on the body mass index (BMI) (Standardized Mean Difference (SMD) = −0.28, 95% CI: −0.72, 0.16) for “non educational settings” and find strongest evidence for primary school-aged children [29]. This finding is limited by the high degree of heterogeneity and the small number of multi-level or environmental change interventions in the underlying studies. In a very recent meta-analysis by Wolfenden and colleagues in which three multi-level interventions and four interventions containing environmental changes were included, a small, significant effect on the age and sex-standardized body mass index (zBMI) in primary school-aged children (Mean Difference (MD) = −0.16, 95% CI: −0.27, −0.05) and a small, non-significant effect for adolescents (MD = −0.02, 95% CI: −0.08, 0.03) were found [30]. In both subgroups heterogeneity was substantial (I^2^ > 90%). In one of the two narrative systematic reviews investigating effects on weight change, Bleich and colleagues found beneficial effects on the children’s BMI or zBMI in four out of nine studies [24]. In the second one, Kellou and colleagues found effects on the children’s BMI, zBMI or fat mass favoring the intervention in all of the five included studies (all multi-level or environmental change interventions) [26]. 

Overall, the results of the last indicate moderate evidence for beneficial effects of community-based interventions on weight change among primary school-aged children, but insufficient evidence for preschool children and adolescents. Among all reviews, only those which included large samples, multi-level and environment change interventions reported beneficial effects. The two reviews by Bleich *et al.* and Waters *et al.* [24,29] provide evidence that including a school component can increase the effectiveness of community-based interventions for primary school-aged children.

### 3.2. General Adult Population

Eight reviews assessed the effectiveness of community-based interventions promoting healthy eating and/ or PA (five meta-analyses [31,32,33,34,35], three narrative systematic reviews [36,37,38]; Table 2). Although this section focusses on the general adult population, some of the reviews also included primary studies that targeted specific groups, such as inactive adults, ethnic minorities, older adults, or women. Unfortunately, in most cases the reviews did not provide separate results for these specific groups. Only one review analyzed effects of community-based interventions on healthy eating [31], eight reviews investigated effects on PA, and none systematically assessed effects on anthropometric measures, such as BMI.

**Table 2:** Selected reviews targeting adult populations.

Author	Type of Review/Type of Interventions Included	Sample/Target Group	Intervention Components	Outcome Measures	Main Results	Evidence and Conclusion
**General adult population: healthy eating**						
Michie *et al.* 2009 [31]	Meta-analysis Not community-based: n = 24 Community recruitment: n = 27 Multi-level intervention: n = 1 Environmental change: n = 1	General adult population or at risk population (obese, low income, women), sample size: 79–3,122	Bag with fresh FV, cookbook, dietary education materials, free cereal servings, tailored print and video material, web-based tailored feedback, lay health visitor support, motivational interviewing counselling, small group seminars, phone calls	FFQ, FV consumption, Fat and Fiber Behavior Questionnaire, macronutrient intake, fat intake, daily grams of fiber, measures converted into SMDs for meta-analysis	Meta-analytic summary of results: positive combined effect for HE (SMD = 0.31, 95% CI: 0.23, 0.39, I^2^ = 73%); no significant difference between community-based studies and studies from other settings; Meta-regression shows that combining self-monitoring with one or more self-regulatory technique improved the effects (SMD = 0.54 *vs.* SMD = 0.24)	Moderate evidence: large number of studies included, but studies of varying quality, outcome assessment and target groups were combined; almost all studies were rather individual-focused. Self-regulation techniques seem to be promising individual-focused approach
**General adult population: physical activity**						
Baker *et al.* 2011 [36]	Narrative systematic review Not community-based: n = 1 Community recruitment: n = 3 Multi-level intervention: n = 13 Environmental change: n = 8	Adult population in high and low income countries, 11 studies provided interventions to deprived areas; sample size: 574–15,261	Local media campaigns, websites, pedometers and logbooks, individual counselling, walking groups, inclusion of specific settings (e.g., shopping malls, churches), community events (fun walks), community task force activities, labelling walk trails	Self-reports of PA: proportions of participants attain a certain level of PA (8 Studies), proportion of inactive or sedentary participants (8 studies), amount of LTPA (3 studies), amount of time spent walking (4 studies), total daily PA (2 studies)	Narrative summary of results: Of 8 studies, only 1 increased the population level to a pre-defined amount of PA; 1 out of 8 studies reported a significant reduction in the proportion of inactive adults favoring the intervention group; Some evidence for an increased amount of LTPA in all 3 studies; Some evidence in 2 out of 4 studies for increased time spent walking; Greater decrease in total daily PA in the comparison area than the intervention area in 1 out of 2 studies; inconclusive results also among more intensive or higher quality studies	Moderate evidence: many community-based studies with adequate sample size included, but only self-reported outcome measures, only 1 RCT included. Authors find insufficient evidence for effectiveness
Ogilvie *et al.* 2007 [37]	Narrative systematic reviewNot community-based: n = 43 Community recruitment: n = 0 Multi-level intervention: n = 3 Environmental change: n = 2	Adults mostly in rural areas, 1 study targeted at sedentary 50- to 65-year-old adults, sample size: 173–1,531	Mass media campaigns, tailored newsletters, walk-a-thons, social support activities, formation of walking groups, park modifications	Self-reports of time spent walking	Narrative summary of results: significant increase in self-reported walking in 2 of 5 studies. Range: −1.4 min/week to +75 min/week, effects were concentrated in most sedentary subgroups	Limited evidence: only self-reported outcome measures and a small number community-based studies included. Evidence based on isolated studies
Kassavou *et al.* 2013 [32]	Meta-analysis Not community-based: n = 2 Community recruitment: n = 17 Multi-level intervention: n = 0 Environmental change: n = 0	Young or middle aged adults (18–59 years, 12 studies), older adults (7 studies), only women (6 studies), sample size: 34–573	Walking groups (lay and expert walk leaders), meeting sessions, self-help material, newsletters	Validated PA questionnaires, pedometers, accelerometers, converted into SMD for meta-analysis	Overall SMD = 0.52 (95% CI: 0.32, 0.71), no significant difference between high and low quality studies; Moderator analysis: stronger effects for interventions targeting both genders *vs.* only women (SMD = 0.61, (95% CI: 0.35, 0.88) *vs.* SMD = .18, (95% CI: 0.03, 0.33), stronger effects among older adults *vs.* younger adults (SMD = 0.57, (95% CI: 0.17, 0.98) *vs.* SMD = 0.48, (95% CI: 0.27, 0.69), no differences between lay and expert walk leaders	Moderate evidence: several small studies included, but large fail-safe N (753 studies), no anthropometric outcomes assessed in the meta-analysis, no complex community-based study included Overall, walking groups seem to be a promising component
Michie *et al.* 2009 [31]	Meta-analysis Not community-based: n = 39 Community recruitment: n = 28 Multi-level intervention: n = 1 Environmental change: n = 1	General adult population or at risk population (sedentary, low activity, obese, at risk for cardiovascular diseases, low income, women), sample size: 37–1,800	Written information, web-based tailored PA monitoring, pedometers, PA diaries, individual counselling, group sessions	Self-reports (mostly validated scales) and pedometer step counts, converted into SMD for meta-analysis	Positive combined effect for PA (SMD = 0.32 (95% CI: 0.26, 0.38), I^2^ = 58%); no significant difference between community-based studies and studies from other settings; Meta-regression shows that combining self-monitoring with one or more self-regulatory technique improved the effects (SMD = 0.38 *vs.* SMD = 0.28)	Moderate evidence: large number of studies included, but studies of varying quality, outcome assessment and target groups were combined; almost all studies were rather individual-focused. Self-regulation techniques seem to be promising individual-focused approach
Garret *et al.* 2011[38]	Narrative systematic review Not community-based: n = 11 Community recruitment: n = 2 Multi-level intervention: n = 0 Environmental change: n = 0	Sedentary adults (18–65 years), inactive couples (28–31 years), sample size: 137–239	Telephone-based feedback on PA, print-based feedback on PA, group sessions, mailed intervention	7- and 14-days PA recall, economic analysis: annual costs per participant to become active, costs of shifting into the active category	Both studies show some evidence for positive PA changes in the low intensity study groups (print-based feedback, mailed intervention); Cost-effectiveness: €884 (print-based feedback) and €3,673 (telephone-based feedback) Costs per QALY: €350 (mailed intervention) and €349 (group sessions)	Limited evidence: small number of studies, no complex community-based intervention, limited effectiveness of the interventions under study
Bock *et al.* 2014 [33]	Meta-analysis Not community-based: n = 0 Community recruitment: n = 48 Multi-level intervention: n = 3 Environmental change: n = 4	General population or inactive adults (23 studies), only women (20 studies), older adults (50+, 22 studies), low SES (8 studies), sample size: 31–3,114	Social marketing, print information material, telephone-only motivational interviewing, face-to-face individual counselling, group education sessions, walking groups, nutrition and PA diaries, pedometers, accelerometers, web-based feedback, social support from community peers, labelling of walking trails, improving street lighting	PA questionnaires (47 studies), step counts derived from pedometers or accelerometers (8 studies), converted into net percent change (NPC) for meta-analysis	Combined effect for PA NPC = 16.4% (95% CI: −6.6%, 39.5%), significant combined effect among high quality studies (16 studies; NPC = 16.2%, 95% CI: 4.4%, 28.0%); subgroup analysis: significant effects if interventions included face-to-face counselling/group sessions (NPC = 35.0%, 95% CI: 9.6%, 60.5%) or mail components (NPC = 18.9%, 95% CI: 2.2%, 35.6%), or if they were focused exclusively on women (NPC = 27.2%, 95% CI: 9.3%, 46.1%)	Moderate evidence: large number of studies included, but of varying quality, significant results only among high quality studies, most studies focused on individual strategies. Inclusion of an individual or group counselling component seems to be a promising component
Webel *et al.* 2010 [34]	Meta-analysis Not community-based: n = 0 Community recruitment: n = 3 Multi-level intervention: n = 1 Environmental change: n = 0	Inactive adults, older adults after myocardial infarction, African-American adults, sample size: 89–725	Lay-led walking groups, lay advisors to spread information and to enhance social support, computerized feedback, lay-led chronic disease self-management course, self-help book	Self-report PA measures, converted into SMD for meta-analysis	Combined SMD in PA = 0.16 (95% CI: 0.05, 0.27) was calculated including 3 studies.	Limited evidence: very few studies included, summary effect includes only 3 out of 5 studies, re-calculation of effects sizes from one primary studies questionable
Soler *et al.* 2010 [35]	Meta-analysis Not community-based: n = 1 Community recruitment: n = 0 Multi-level intervention: n = 0 Environmental change: n = 12	General population in public spaces (e.g., shopping mall, train stations, libraries), sample size: 12,288–158,350 observations	Signs encouraging stair use posted on wall next to stair areas and elevator, vinyl footprints stuck on floor leading to stairs, enhancements to stairwells (carpets, artwork, music, paintings)	Frequency of stair use recorded, converted into absolute (percentage points) and relative change in stair use	Median absolute increase in stair use of 2.4 percentage points (IQI: 0.8, 6.7), median relative improvement: 50% (IQI: 5.4, 90.6); insufficient evidence for motivational signs plus stairwell enhancements	Moderate evidence: large observational studies included, but no effects on overall PA, no RCTs included. Overall, using motivational signs seems to be a promising strategy
**Adults at risk population: socially disadvantaged women**						
Cleland *et al.* 2012 [39]	Meta-analysis Not community-based: n = 1 Community recruitment: n = 16 Multi-level intervention: n = 2 Environmental change: n = 0	Socially disadvantaged adult women (18–64 years), sample size: 43–1,578	Print information material on HE and PA benefits, group education sessions, pedometer feedback, computer-tailored messages, telephone counselling, text messages, exercise lessons, written information on walking routes	Self-reported PA (16 studies), pedometer or accelerometer (3 studies), converted into SMD for meta-analysis	No pooled effect computed due to high degree of heterogeneity, the authors; subgroup analysis: interventions were more effective if they included a group component (SMD = 0.36, 95% CI: 0.17, 0.54), community interventions were effective if they were placed in community organization (e.g., churches, SMD = 0.26, 95% CI: 0.03, 0.49).	Moderate evidence: large number of studies included
**Adults at risk population: adults with prediabetes**						
Norris *et al.* 2009 [40]	Meta-analysis Not community-based: n = 4 Community recruitment: n = 5 Multi-level intervention: n = 0 Environmental change: n = 0	Adults with impaired glucose tolerance, sample size: 88–574	Counselling or encouraging to increase PA or HE, supervised activity sessions, exercise diaries, stress management, residential treatment	HE: not assessed in the review PA: not assessed in the review Weight: weight change in kg Other: diabetes incidence	Pooled effect for weight change = −2.6 kg (95% CI: −3.3 to −1.9) at 2-year FU. Decrease in diabetes incidence in 1 of 3 studies (58% RR reduction).	Moderate evidence: sufficient sample size and valid outcome parameters, but no multi-level or environmental change intervention included
Baker P *et al.* 2011 [41]	Narrative systematic review Not community-based: n = 0 Community recruitment: n = 5 Multi-level intervention: n = 0 Environmental change: n = 0	Adults with impaired glucose tolerance, overweight, adults, sample size: 325–3,234	Individual counseling, supervised exercise sessions, progressive resistance training, individual goal setting	HE: not assessed in the review PA: not assessed in the review Weight: not assessed in the review Other: diabetes incidence	RR reduction in diabetes incidence ranged from 29%–75% Interventions with theory-based behavioral strategies were more effective than information and advice approaches	Moderate evidence: sufficient sample size and valid outcome parameters, but no multi-level or environmental change intervention included

Notes: BMI: body mass index, CI: confidence interval, FFQ: food frequency questionnaire, FV: fruit and vegetable, FU: follow up, HE: healthy eating, IQI: interquartile interval, LTPA: leisure time physical activity, NPC: net percent change, PA: physical activity, QALY: quality-adjusted life year, RR: relative risk, SMD: standardized mean difference, T2D: type II diabetes.

#### 3.2.1. Effectiveness of Interventions on Healthy Eating

The meta-analysis by Michie and colleagues provides moderate evidence for the effectiveness of community-based interventions on healthy eating [31]. They report a positive combined effect for healthy eating derived from various food frequency questionnaires across all studies (SMD = 0.31, 95% CI: 0.23, 0.39, I^2^ = 73%) with no significant difference between community-based studies and studies from other settings (workplace, healthcare settings). The evidence from this review is limited by the fact that almost no multi-level or environmental change intervention was included. In addition, studies of varying quality were pooled and substantial heterogeneity was reported.

The authors investigated a list of 26 potential behavior change strategies, e.g., providing general information, modeling behavior, setting graded tasks, feedback on performance, and analyzed whether any of these techniques was associated with effectiveness. The results from the meta-regression indicate that combining self-monitoring with one or more self-regulatory techniques improved the effects on healthy eating (SMD = 0.54 *vs.* SMD = 0.24). 

#### 3.2.2. Effectiveness of Interventions on Physical Activity

In the aforementioned meta-analysis, Michie and colleagues also report a pooled beneficial effect of community-based interventions on PA derived from questionnaire and objective PA data (SMD = 0.32, 95% CI: 0.26, 0.38, I^2^ = 58%). Once again, the evidence was limited by the lack of multi-level or environmental change interventions and the pooling of studies of differing quality. According to the meta-regressive results, combining self-monitoring with one or more self-regulatory technique improved the effects on PA (SMD = 0.38 *vs.* SMD = 0.28). 

The narrative systematic review by Baker and colleagues focuses on community-wide interventions and incorporates a large proportion of multi-level and environmental change intervention with moderate to large sample sizes. The authors assessed a variety of self-reported PA outcome measures and did not find sufficient evidence for the effectiveness of community-based interventions. None of the included studies was classified as being at a low risk of bias because all of them did not use random allocation of the study groups or an objective measure of PA [36]. 

A further review from Ogilvie and colleagues focused on interventions to promote walking [37]. The review comprised three multi-level interventions and environmental change interventions including mass media campaigns, walking groups, community events, and park modifications. Of the five studies, two showed a significant net increase in self-reported time spent walking at 12 months follow-up. The authors concluded that the evidence was still insufficient as the review included only a small number of community-based studies. 

A recent meta-analysis by Bock and colleagues included 55 intervention studies (seven multi-level or environmental change interventions) [33]. The authors found a significant net percent change (NPC) in PA (both self-report and objective measures) of 16% (95% CI: 4.4%, 28.0%) among high quality studies (16 studies). Subgroup analysis indicated significant effects if interventions included face-to-face counselling/group sessions (NPC = 35.0%, 95% CI: 9.6%, 60.5%) or mail components (NPC = 18.9%, 95% CI: 2.2%, 35.6%), or if they were focused exclusively on women (NPC = 27.2%, 95% CI 9.3%, 46.1%).

According to Webel *et al.*, a peer-based intervention strategy can be defined as a method in which people share specific health messages with members of their community [34]. With regard to community-based interventions, Webel and colleagues included four intervention studies that mostly applied lay-led walking groups or counselling sessions. The authors found some evidence for effectiveness based on a meta-analysis including three studies of peer interventions. Although a significant increase in self-reported PA (SMD = 0.16, 95% CI: 0.05, 0.27) was reported, the small number of studies precluded strong conclusions on evidence.

In a recent meta-analysis including 17 community recruitment studies, Kassavou and colleagues investigated the effect of walking groups on PA behavior [32]. The authors converted validated self-report and objective PA data into standardized mean differences and found a pooled beneficial effect on PA of overall SMD = 0.52 (95% CI: 0.32, 0.71), with no significant difference between high and low quality studies. Subgroup analyses indicated stronger effects for interventions targeting both genders compared to interventions targeting only women, and stronger effects among older adults compared to younger adults. No differences occurred between interventions using lay and expert walk leaders.

One review investigated the effect of point-of-decision prompts on stair use based on 11 environmental change intervention studies [35]. The authors included diverse settings such as shopping malls and train stations, and reported that motivation signs led to a small but significant increase in the proportion of people using the stairs (2.7 percentage points). A combination of motivational signs and stairwell enhancement however did not result in stronger effects. The studies focused on stair use as an outcome and did not consider overall PA. 

One review analyzed the cost-effectiveness of PA interventions but included only two community-based interventions [38]. The cost-effectiveness to move a person into the active category at 12 months (€884 and €3,673) and cost per quality-adjusted life year (QALY) (€349 and €350) of the community interventions, however, indicated a good cost-benefit ratio. The authors state that most of the PA interventions were below the acceptable threshold for funded interventions, considered to lie between ₤20,000 to ₤30,000 as reported by the UK National Institute for Health and Clinical Excellence [42]. Nevertheless, the evidence for cost-effectiveness or cost-utility is very limited due to the small number of informative studies available.

### 3.3. Adult at Risk Populations

#### 3.3.1. Socially Disadvantaged Women

Assessing the effectiveness of PA community-based interventions targeted at socially disadvantaged women, Cleland and colleagues included 18 community-based studies with few multi-level and no environmental change interventions [39]. Due to high degrees of heterogeneity, the authors did not provide a pooled effects size, but they report subgroup effects. The results indicate that interventions were more effective if they included a group component (SMD = 0.36, 95% CI: 0.17, 0.54). Furthermore, community interventions were effective if they were delivered by community organizations (e.g., churches, SMD = 0.26, 95% CI: 0.03, 0.49).

#### 3.3.2. Adults at Risk for Type II Diabetes

Two reviews included studies focusing on adults at risk for type II diabetes [40,41]. Both reviews report beneficial effects in weight change or BMI as well as on diabetes incidence, with a relative risk reduction of 29%–75% for the latter. In sum, it appears that while the evidence for effectiveness in the general adult population is still disputable, evidence exists for community interventions focusing on adults at risk for type II diabetes. The two reviews that addressed adult populations at risk for diabetes found that the most successful interventions in their meta-analysis had adopted an intensive, long-term approach involving several intervention components. Specifically, Norris et al report a significant correlation between the number of intervention contacts and decrease in weight. In general, Baker *et al.* observed that interventions that included behavior change strategies and were theory-based were more effective than the information and advice approaches given to the control group [27]. As both reviews did not include multi-level or environmental change interventions, the evidence from these studies is limited to individual-focused approaches in the community.

### 3.4. Discussion

In this review of reviews, we investigated the effectiveness of community-based interventions to promote PA and healthy eating. Specifically, our goal was to identify promising intervention strategies. Overall, 18 reviews of moderate to good methodological quality according to the AMSTAR criteria were included. The included reviews differed with regard to the target groups of the underlying studies (children and adolescents, general adult population, and specific adult at risk groups), the outcomes assessed (healthy eating, PA, weight change and other anthropometric measures), and the types of community-based interventions (community recruitment, multi-level, and environmental change) included. 

With regard to children and adolescents, the reviews did not provide evidence for beneficial effects on healthy eating or PA. However, there was moderate evidence from three of the seven reviews [24,26,30] for beneficial effects of community-based interventions on weight change. Compared to the reviews reporting no effects, these reviews included primary studies with larger sample sizes and more studies addressing multiple social-ecological levels or environmental changes. It seems that more comprehensive community-based approaches are more successful in targeting weight change in children and adolescents. Moreover, the reviews indicated that the evidence for beneficial effects was strongest for primary school-aged children and insufficient for adolescents and preschool children. In the latter case, only a small number of primary studies was available [29,30]. A combination of school components, such as more and enhanced PA lessons at school, changes of the food environment at school, and community-based approaches (e.g., awareness campaigns, parent counselling, community capacity building) is indicated as a promising strategy in two of the reviews [24,29].

Reviews on community-based interventions targeting the general adult population provided equivocal conclusions. None of the reviews assessed anthropometric measures, such as BMI, and only one review analyzed intervention effects on healthy eating finding moderate evidence for beneficial effects [31]. However, the interventions included in this review mostly applied a community recruitment approach with individual-focused intervention strategies, such as provision of (tailored) information material, individual or group counselling, and pedometers for self-monitoring. Assessing effective behavior chance strategies, this review provided evidence that self-monitoring (in combination with additional strategies derived from the behavior regulation theory) contributes to intervention effectiveness.

All reviews targeting general adult population assessed PA. Two meta-analyses including a large number of studies showed evidence for moderate effects of community-based intervention on PA [31,33]. However, the review by Baker and colleagues reported insufficient evidence for beneficial effects of communities on PA. Differences between the two reviews with regard to selection criteria and operationalization of the term “community-based intervention” may explain why the reviews came to different conclusions. For example, from the 25 studies in the review by Baker *et al.* [36], only one was included in the meta-analysis by Michie *et al.* [31]. While the two meta-analyses primarily included interventions applying community recruitment approaches, most interventions in the review of Baker *et al.* targeted multiple levels or environmental change strategies. There are several potential reasons for the inconclusive effectiveness of more comprehensive community-based intervention that address the general population. Specifically, low levels of community penetration and exposure to health promotion activities have been discussed to be major reasons [19].

Although the overall evidence for beneficial effects is still inconclusive, some of the other included reviews gave hints on promising interventions strategies. At the environmental level, motivational signs have been identified to increase stair use, although the evidence for this strategy was limited by the fact that overall PA was not taken into account and no study applied random allocation. On the interpersonal level, Webel *et al.* [34] provided some evidence for the effectiveness of peer-based PA interventions (mostly lay-led walking groups), and there is moderate evidence that walking groups (lay-led or expert-led) are an effective intervention component to increase PA [32]. A group component was also found to be effective in two other reviews [33,39]. On the individual level, as for healthy eating outcome, Michie *et al.* found increased effects on PA if the interventions applied self-regulation behavior change techniques [31]. A reasonable intervention strategy may be to combine the different promising components into one community-based intervention approach.

With regard to adult at risk population, we included one review on interventions promoting PA among socially disadvantaged women and two reviews targeting adults at risk for type II diabetes. Cleland *et al.* included interventions that used community recruitment to target socially disadvantaged women [39]. They found that interventions using a group component and those delivered by community organizations were more effective. Other reviews also reported gender differences. One review found that walking group interventions were wore effective if both genders targeted were targeted [32]. In contrast to that one other review reported stronger effects for PA interventions that focused exclusively on women [33]. Thus, the benefits of gender-specific PA interventions seem inconclusive.

Two reviews on adults at risk for type II diabetes provided evidence that combined healthy eating and interventions can effectively contribute to weight loss and reduce the risk for type II diabetes in at-risk adults [40,41]. The authors found stronger effects for intensive, long term interventions. None of reviews included multi-level or environmental change strategies. The effectiveness of the interventions within broader community intervention contexts is subject to future research. 

A strength of this review of reviews is that it includes systematic reviews in which only primary studies that applied an experimental (randomized controlled trial) or quasi-experimental study design (non-random control group, interrupted time series), were included *i.e.*, the validity of the study designs was comparatively high. However, this approach led to the exclusion of a considerable number of reviews and studies with weaker designs. We note that it is often hard to find adequate control communities when evaluating the community-based interventions. This especially applies to interventions where environmental changes are planned or conducted [20]. Even though we introduced above restrictions, the application of the AMSTAR tool was not always straight forward, as information was missing and some items seemed not well suited for the body of reviews we studied. Additional critical appraisal of the underlying studies was necessary to adequately assess the evidence presented in the reviews.

Although the 18 included reviews summarized results of 196 primary studies, several of them included only a small number of community-based interventions, which limited our ability to draw valid conclusions from these reviews. Furthermore, the primary studies were very heterogeneous with regard to the extent to which the whole community was addressed. In many cases the community was treated as a setting for recruiting participants or delivering the intervention. The interventions entailed changes in the communities’ social and physical surroundings only in a few cases. The evidence for environmental changes to increase PA and healthy eating is hence still very limited. The reviews generally reported several methodological weaknesses of the included complex community interventions. Most of the studies included only a small number of communities (mostly one intervention and one control community, see [36]). To reach an adequate statistical power it has been estimated that at least ten communities per study condition are necessary [43]. In addition, most of the studies relied on self-report measures of PA and healthy eating and did not include objective measures (e.g., accelerometers). There was also a lack of information with regard to intervention reach and fidelity of implementation. This information is necessary to estimate the population-level impact of the intervention and may also help to explain why effectiveness was not achieved among healthy adults [19]. 

In this review of reviews we identified a small number of promising intervention strategies. Future research should generate more multi-level or environmental change intervention studies that apply high-quality research designs with objectively measured outcomes, have sufficient statistical power and include indicators for reach and fidelity. From our perspective, more studies that test different intervention strategies against each other in order to generate more evidence on effective strategies are needed. In addition, recent research has highlighted the important influence of environments, including the concept of neighborhood walkability and the community availability of high-fat, sugar-rich foods [44,45]. Insights from this area of research should inspire future intervention development of community-based interventions. For example, Giles-Corti and colleagues investigated the impact of relocating people to a new “walkable” urban housing development in a natural experiment [46]. More studies of this kind which investigate the effects of major environmental changes on PA and healthy eating are required. However, the challenges to such work are extensive.

## 4. Conclusions

Community-based interventions for health promotion and prevention are important approaches for public health, but the evaluation of these interventions is associated with numerous methodological challenges. In this review of reviews we found moderate evidence that community-based interventions can have beneficial effects on weight gain among primary school-aged children. A combination of school-based and community-based interventions is a promising strategy. Evidence for community-based interventions targeting the general adult population is limited by the lack of multi-level or environmental change intervention studies using objective PA or anthropometric outcome measures. Self-monitoring elements, (walking) group components and point-of-decision prompts to use stairs are promising interventions strategies to address different social-ecological levels. Combined healthy eating and PA interventions can contribute to weight loss and reduce the risk for type II diabetes in adults with prediabetes. However, these approaches have not been assessed in a more comprehensive community-based approach. More studies that investigate the effects of changing environments on population health and health behavior are needed. 

## Supplementary Files

Supplementary File 1 Supplementary Information (PDF, 206 KB)
