# Supplementary material for: What Works in Community-Based Interventions Promoting Physical Activity and Healthy Eating? A Review of Reviews
Source: Int J Environ Res Public Health. 2014 May 30;11(6):5866–88. doi: 10.3390/ijerph110605866 (PMC4078553; doi:10.3390/ijerph110605866)
Supplement: Supplementary File 1 — Supplementary Information (PDF, 206 KB) [file ijerph-11-05866-s001.pdf]

Supplementary Information

What Works in Community-Based Interventions Promoting Physical Activity and Healthy Eating? A Review of Reviews

---

Search Protocol

Update: 14 April 2014

Limits: publication date 2007–present, publication type "review", publication language "English".

1. (((((((systematic review or meta analysis[Publication Type])) AND ("2007"[Date-Publication]: "3000"[Date-Publication])) AND (prevention or intervention or promot* [Title/Abstract])) AND (community or neighborhood or quarter or population-based- or multi level or multi-component or environmental intervention or social environment or built environment)) AND (dietary or weight or nutrition or healthy eating or body mass index or obese or obesity or vegetable or fruit or overweight)))
2. ((((((systematic review or meta analysis[Publication Type])) AND ("2007"[Date-Publication]: "3000"[Date-Publication])) AND (prevention or intervention or promot* [Title/Abstract])) AND (community or neighborhood or quarter or population-based- or multi level or multi-component or environmental intervention or social environment or built environment)) AND (physical activity or physical inactivity or motor activity or exercise or ergonomic or musculoskeletal disorder or fitness or sedentary behav*))
3. (1 or 2)

Hits: 2,091 → filters activated: 1,819

NICE:

Searched: 14 April 2014

Limits: areas of interest "Public Health", types of information "systematic review", date "2007–present", Sources "DARE", "NICE", "NIHR Journals Library"

((prevention or intervention or promot*) and (community or neighborhood or quarter or population-based- or multi level or multi-component or environmental intervention or social environment or built environment) and (dietary or weight or nutrition or healthy eating or body mass index or obese or obesity or vegetable or fruit or overweight)) or ((prevention or intervention or promot*) and (community or neighborhood or quarter or population-based- or multi level or multi-component or environmental intervention or social environment or built environment) and (physical activity or physical inactivity or motor activity or exercise or ergonomic or musculoskeletal disorder or fitness or sedentary behave*))

Hits: 148

Cochrane:

Searched: 14th April 2014

Limits: publication date: 2007–present, Database “Cochrane Reviews (Reviews only) and Other Reviews”.

1. prevention or intervention or promot*: ti,ab,kw and community or neighborhood or quarter or population-based- or multi level or multi-component or environmental intervention or social environment or built environment:ti,ab,kw and dietary or weight or nutrition or healthy eating or body mass index or obese or obesity or vegetable or fruit or overweight: ti,ab,kw Publication Date from 2007, in Other Reviews (Word variations have been searched)
2. prevention or intervention or promot*: ti,ab,kw and community or neighborhood or quarter or population-based- or multi level or multi-component or environmental intervention or social environment or built environment:ti,ab,kw and physical activity or physical inactivity or motor activity or exercise or ergonomic or musculoskeletal disorder or fitness or sedentary behave *: ti,ab,kw Publication Date from 2007, in Other Reviews (Word variations have been searched)
3. (1 or 2)

Hits: 113

Campbell:

Searched: 14 April 2014

Limits: publication date 2007–present, document type “review”

1. prevention OR intervention OR promot* in all text and community OR neighborhood OR quarter OR population-based- OR multi level OR multi-component OR environmental intervention OR social environment OR built environment in all text and dietary OR weight OR nutrition OR healthy eating OR body mass index OR obese OR obesity OR vegetable OR fruit OR overweight in all text and published from 2007 and document type is review
2. prevention OR intervention OR promot* in all text and community OR neighborhood OR quarter OR population-based- OR multi level OR multi-component OR environmental intervention OR social environment OR built environment in all text and physical activity OR physical inactivity OR motor activity OR exercise OR ergonomic OR musculoskeletal disorder OR fitness OR sedentary behav* in all text and published from 2007 and document type is review
3. (1 or 2)

Hits: 73

Two authors independently assessed the quality of all selected reviews according to the AMSTAR criteria, an 11-item questionnaire developed to assess the methodological quality of systematic reviews. Table S1 shows the results of the quality rating. In the last column, number of positive rating (“Yes”) was summed up for an overall quality score of the reviews.

Table S1. Quality assessment of the included reviews.

Author	Review Type	#1 a Priori Design?	#2 Duplicate Selection?	#3 Comprehensive Search?	#4 Grey Literature?	#5 List of Studies Provided?	#6 Study Characteristics Provided?	#7 Quality Critically Assessed?	#8 Enough Quality for Conclusion?	#9 Results Appropriately Combined?	#10 Publication Bias Assessed ?	#11 Conflict of Interests Assessed?	Score
Bleich *et al.*, 2013 [1] *	Narrative	Yes	Yes	Yes	Yes	Yes	Yes	Yes	Yes	Not applicable	No	No	8
Hendrie *et al.*, 2011 [2]	Narrative	Yes	Can't answer	yes	No	Yes	Yes	Yes	Yes	Not applicable	No	No	6
Kellou *et al.*, 2014 [3]	Narrative	Yes	Yes	Yes	No	Yes	Yes	Yes	Yes	Not applicable	No	No	7
van Sluijs *et al.*, 2007 [4]	Narrative	Yes	Yes	Yes	No	Yes	Yes	Yes	Yes	Not applicable	no	No	7
Van Sluijs *et al.*, 2011 [5]	Narrative	Yes	No	No	No	Yes	Yes	Yes	Yes	Not applicable	No	No	5
Waters *et al.*, 2011 [6]	Meta-analysis	Yes	Yes	Yes	Yes	Yes	Yes	Yes	Yes	Not applicable	Yes	No	9
Wolfenden *et al.*, 2014 [7]	Meta-analysis	Yes	Yes	Yes	Yes	Yes	Can't answer	Yes	Yes	Yes	No	No	9
Michie *et al.*, 2009 [8]	Meta-analysis	Yes	Yes	Yes	No	Yes	No	No	No	Yes	Yes	No	6

Table S1. *Cont.*

Author	Review Type	#1 a Priori Design?	#2 Duplicate Selection?	#3 Comprehensive Search?	#4 Grey Literature?	#5 List of Studies Provided?	#6 Study Characteristics Provided?	#7 Quality Critically Assessed?	#8 Enough Quality for Conclusion?	#9 Results Appropriately Combined?	#10 Publication Bias Assessed ?	#11 Conflict of Interests Assessed?	Score
Kassavou *et al.*, 2013 [9]	Meta-analysis	Yes	No	Yes	Yes	Yes	Yes	Yes	Yes	Yes	No	No	8
Bock *et al.*, 2014 [10]	Meta-analysis	Yes	Can't answer	Yes	No	Yes	No	Yes	Yes	Yes	No	No	6
Webel *et al.*, 2010 [11]	Meta-analysis	Yes	No	Can't answer	No	Yes	No	Yes	Yes	Yes	Yes	No	6
Soler *et al.*, 2010 [12]	Meta-analysis	Yes	Can't answer	Yes	Can't answer	Yes	No	Yes	Yes	Not applicable	No	No	5
Baker *et al.*, 2011 [13]	Narrative	Yes	Yes	Yes	Yes	Yes	Yes	Yes	Yes	No	No	No	8
Ogilvie *et al.*, 2007 [14]	Narrative	Yes	Yes	Yes	Yes	Yes	Yes	Yes	Can't answer	Not applicable	Can't answer	No	7
Garret *et al.*, 2011 [15]	Narrative	Yes	No	Can't answer	No	Yes	Yes	Yes	Yes	Not applicable	No	No	5
Cleland *et al.*, 2012 [16]	Meta-analysis	Yes	Yes	Yes	No	Yes	Yes	Yes	Yes	Yes	No	No	9
Norris *et al.*, 2009 [17]	Meta-analysis	Yes	No	Yes	Yes	Yes	Yes	Yes	Yes	Yes	Yes	No	9
Baker *et al.*, 2011 [18]	Narrative	Yes	Can't answer	Yes	Yes	Yes	Can't answer	Yes	Yes	Not applicable	No	No	6

Note: * After removal of duplicates, the 18 selected reviews included 195 primary studies (see section "Reference List of the Underlying Primary Studies") of community-based interventions promoting physical activity and healthy eating. The following list shows the references of the underlying studies.

Reference List of the Underlying Primary Studies

- Adams, J.; White, M. A systematic approach to the development and evaluation of an intervention promoting stair use. *Health Educ. J.* **2002**, *61*, 272–286.
- Ahluwalia, J.S.; Nollen, N.; Kaur, H.; James, A.S.; Mayo, M.S.; Resnicow, K. Pathway to health: Cluster-randomized trial to increase fruit and vegetable consumption among smokers in public housing. *Health Psychol.* **2007**, *26*, 214–221.
- Albright, C.L.; Pruitt, L.; Castro, C.; Gonzalez, A.; Woo, S.; King, A.C. Modifying physical activity in a multiethnic sample of low-income women: One-year results from the IMPACT (Increasing Motivation for Physical ACTivity) project. *Ann. Behav. Med.* **2005**, *30*, 191–200.
- Aldana, S.G.; Greenlaw, R.L.; Diehl, H.A.; Salberg, A.; Merrill, R.M.; Ohmine, S.; Thomas, C. Effects of an intensive diet and physical activity modification program on the health risks of adults. *J. Amer. Diet. Assn*. **2005**, *105*, 371–381.
- Andersen, R.E.; Franckowiak, S.C.; Snyder, J.; Bartlett, S.J.; Fontaine, K.R. Can inexpensive signs encourage the use of stairs? Results from a community intervention. *Ann. Intern. Med.* **1998**, *129*, 363–369.
- Andersen, R.E.; Franckowiak, S.C.; Zuzak, K.B.; Cummings, E.S.; Crespo, C.J. Community intervention to encourage stair use among african-american commuters. *Med. Sci. Sport. Exercise* **2000**, *32*, S38.
- Anderson, D.; Mizzari, K.; Kain, V.; Webster, J. The effects of a multimodal intervention trial to promote lifestyle factors associated with the prevention of cardiovascular disease in menopausal and postmenopausal australian women. *Health Care Women Int*. **2006**, *27*, 238–253.
- Anderson, E.S.; Winett, R.A.; Wojcik, J.R.; Winett, S.G.; Bowden, T. A computerized social cognitive intervention for nutrition behavior: Direct and mediated effects on fat, fiber, fruits, and vegetables, self-efficacy, and outcome expectations among food shoppers. *Ann. Behav. Med*. **2001**, *23*, 88–100.
- Armitage, C.J. Effects of an implementation intention-based intervention on fruit consumption. *Psychol. Health* **2007**, *22*, 917–928.
- Ash, S.; Reeves, M.; Bauer, J.; Dover, T.; Vivanti, A.; Leong, C.; O'Moore Sullivan, T.; Capra, S. A randomised control trial comparing lifestyle groups, individual counselling and written information in the management of weight and health outcomes over 12 months. *Int. J. Obesity* **2006**, *30*, 1557–1564.
- Backman, D.; Scruggs, V.; Atiedu, A.A.; Bowie, S.; Bye, L.; Dennis, A.; Hall, M.; Ossa, A.; Wertlieb, S.; Foerster, S.B. Using a toolbox of tailored educational lessons to improve fruit, vegetable, and physical activity behaviors among African American women in California. *J. Nutr. Educ. Behav.* **2011**, *43*, S75–S85.
- Baker, G.; Gray, S.R.; Wright, A.; Fitzsimons, C.; Nimmo, M.; Lowry, R.; Mutrie, N.; Scottish Physical Activity Research Collaboration (SPARColl). The effect of a pedometer-based community walking intervention “Walking for Wellbeing in the West” on physical activity levels and health outcomes: A 12-week randomized controlled trial. *Int. J. Behav. Nutr. Phys. Act*. **2008**, *5*, doi:10.1186/1479-5868-5-44.

- Ball, K.; Salmon, J.; Leslie, E.; Owen, N.; King, A.C. Piloting the feasibility and effectiveness of print- and telephone-mediated interventions for promoting the adoption of physical activity in Australian adults. *J. Sci. Med. Sport* **2005**, *8*, 134–142.
- Banks-Wallace, J. Outcomes from walk the talk: A nursing intervention for black women. *ABNF J.* **2007**, *18*, 19–24.
- Baranowski, T.; Baranowski, J.C.; Cullen, K.W.; Thompson, D.I.; Nicklas, T.; Zakeri, I.E.; Rochon, J. The Fun, Food, and Fitness Project (FFFP): The baylor gems pilot study. *Ethn. Dis.* **2003**, *13*, S30–S39.
- Baranowski, T.; Simons-Morton, B.; Hooks, P.; Henske, J.; Tiernan, K.; Dunn, J.K.; Burkhalter, H.; Harper, J.; Palmer, J. A center-based program for exercise change among Black-American families. *Health Educ. Quart.* **1990**, *17*, 179–196.
- Bennett, J.A.; Young, H.M.; Nail, L.M.; Winters-Stone, K.; Hanson, G. A telephone-only motivational intervention to increase physical activity in rural adults: A randomized controlled trial. *Nurs. Res.* **2008**, *57*, 24–32.
- Black, M.M.; Hager, E.R.; Le, K.; Anliker, J.; Arteaga, S.S.; Diclemente, C.; Gittelsohn, J.; Magder, L.; Papas, M.; Snitker, S. *et al.* Challenge! Health promotion/obesity prevention mentorship model among urban, black adolescents. *Pediatrics* **2010**, *126*, 280–288.
- Blamey, A.; Mutrie, N.; Aitchison, T. Health promotion by encouraged use of stairs. *BMJ* **1995**, *311*, 289–290.
- Bo, S.; Ciccone, G.; Baldi, C.; Benini, L.; Dusio, F.; Forastiere, G.; Lucia, C.; Nuti, C.; Durazzo, M.; Cassader, M. *et al.* Effectiveness of a lifestyle intervention on metabolic syndrome. A randomized controlled trial. *J. Gen. Intern. Med.* **2007**, *22*, 1695–1703.
- Bolognesi, M.; Nigg, C.R.; Massarini, M.; Lippke, S. Reducing Obesity Indicators through Brief Physical Activity Counseling (PACE) in Italian primary care settings. *Ann. Behav. Med.* **2006**, *31*, 179–185.
- Boutelle, K.N.; Jeffery, R.W.; Murray, D.M.; Schmitz, M.K. Using signs, artwork, and music to promote stair use in a public building. *Amer. J. Public Health* **2001**, *91*, 2004–2006.
- Brown, W.J.; Mummery, K.; Eakin, E.G.; Schofield, G.M. 10,000 steps rockhampton: evaluation of a whole community approach to improving population levels of physical activity. *J. Phys. Act. Health* **2006**, *3*, 1–14.
- Brown, W.J.; Lee, C.; Oyomopito, R. Effectiveness of a bilingual heart health program for Greek-Australian women. *Health Promot. International* **1996**, *11*, 117–125.
- Brownell, K.D.; Stunkard, A.J.; Albaum, J.M. Evaluation and modification of exercise patterns in the natural environment. *Amer. J. Psychiat.* **1980**, *137*, 1540–1545.
- Brownson, R.C.; Baker, E.A.; Boyd, R.L.; Caito, N.M.; Duggan, K.; Housemann, R.A.; Kreuter, M.W.; Mitchell, T.; Motton, F.; Pulley, C.; *et al.* A community-based approach to promoting walking in rural areas. *Amer. J. Prev. Med.* **2004**, *27*, 28–34.
- Brownson, R.C.; Hagood, L.; Lovegreen, S.L.; Britton, B.; Caito, N.M.; Elliott, M.B.; Emery, J.; Haire-Joshu, D.; Hicks, D.; Johnson, B.; *et al.* A multilevel ecological approach to promoting walking in rural communities. *Prev. Med.* **2005**, *41*, 837–842.

- Brug, J.; Glanz, K.; Van Assema, P.; Kok, G.; van Breukelen, G.J. The impact of computer-tailored feedback and iterative feedback on fat, fruit, and vegetable intake. *Health Educ. Behav.* **1998**, *25*, 517–531.
- Buman, M.P.; Giacobbi, P.R., Jr.; Dzierzewski, J.M.; Aiken Morgan, A.; McCrae, C.S.; Roberts, B.L.; Marsiske, M. Peer volunteers improve long-term maintenance of physical activity with older adults: A randomized controlled trial. *J. Phys. Act. Health* **2011**, *8*, S257–S266.
- Burke, V.; Giangiulio, N.; Gillam, H.F.; Beilin, L.J.; Houghton, S. Physical activity and nutrition programs for couples: A randomized controlled trial. *J. Clin. Epidemiol.* **2003**, *56*, 421–432.
- Campbell, M.K.; Carbone, E.; Honess-Morreale, L.; Heisler-MacKinnon, J.; Farrell, D.; Demissie, S. Development and evaluation of a multimedia tailored nutrition education program for women. *J. Nutr. Educ. Behav.* **2004**, *36*, 58–66.
- Campbell, M.K.; DeVellis, B.M.; Strecher, V.J.; Ammerman, A.S.; DeVellis, R.F.; Sandler, R.S. Improving dietary behavior: The effectiveness of tailored messages in primary care settings. *Amer. J. Public Health* **1994**, *84*, 783–787.
- Campbell, M.K.; Honess-Morreale, L.; Farrell, D.; Carbone, E.; Brasure, M. A tailored multimedia nutrition education pilot program for low-income women receiving food assistance. *Health Educ. Res.* **1999**, *14*, 257–267.
- Campbell, M.K.; James, A.; Hudson, M.A.; Carr, C.; Jackson, E.; Oakes, V.; Demissie, S.; Farrell, D.; Tessaro, I. Improving multiple behaviors for colorectal cancer prevention among African American church members. *Health Psychol* **2004**, *23*, 492–502.
- Carroll, D.L.; Rankin, S.H. Comparing interventions in older unpartnered adults after myocardial infarction. *Eur. J. Cardiovasc. Nurs.* **2006**, *5*, 83–89.
- Chang, M.W.; Nitzke, S.; Brown, R. Design and outcomes of a mothers in motion behavioral intervention pilot study. *J. Nutr. Educ. Behav.* **2010***, 42*, S11–S21.
- Coleman, K.J.; Gonzalez, E.C. Promoting stair use in a U.S.-Mexico border community. *Amer. J. Public Health* **2001**, *91*, 2007–2009.
- Costanzo, C.; Walker, S.N.; Yates, B.C.; McCabe, B.; Berg, K. Physical activity counseling for older women. *West. J. Nurs. Res.* **2006**, *28*, 786–801.
- Coull, A.J.; Taylor, V.H.; Elton, R.; Murdoch, P.S.; Hargreaves, A.D. A randomised controlled trial of senior lay health mentoring in older people with ischaemic heart disease: The braveheart project. *Age Ageing* **2004**, *33*, 348–354.
- Cox, K.L.; Burke, V.; Gorely, T.J.; Beilin, L.J.; Puddey, I.B. Controlled comparison of retention and adherence in home- *vs.* center-initiated exercise interventions in women ages 40–65 years: The S.W.E.A.T. Study (Sedentary Women Exercise Adherence Trial). *Prev. Med.* **2003**, *36*, 17–29.
- Croteau, K.A.; Richeson, N.E.; Farmer, B.C.; Jones, D.B. Effect of a pedometer-based intervention on daily step counts of community-dwelling older adults. *Res. Quart. Exercise Sport* **2007**, *78*, 401–406.
- De Bourdeaudhuij, I.; Brug, J. Tailoring dietary feedback to reduce fat intake: An intervention at the family level. *Health Educ. Res.* **2000**, *15*, 449–462.
- De Cocker, K.A.; De Bourdeaudhuij, I.M.; Brown, W.J.; Cardon, G.M. Four-year follow-up of the community intervention "10,000 steps ghent". *Health Educ. Res.* **2011**, *26*, 372–380.

- De Cocker, K.A.; de Bourdeaudhuij, I.M.; Brown, W.J.; Cardon, G.M. Effects of "10,000 steps ghent": A whole-community intervention. *Amer. J. Prev. Med.* **2007**, *33*, 455–463.
- De Cocker, K.A.; de Bourdeaudhuij, I.M.; Cardon, G.M. The effect of pedometer use in combination with cognitive and behavioral support materials to promote physical activity. *Patient Educ. Couns*. **2008**, *70*, 209–214.
- De Jong, J.; Lemmink, K.A.; Stevens, M.; de Greef, M.H.; Rispens, P.; King, A.C.; Mulder, T. Six-month effects of the Groningen Active Living Model (GALM) on physical activity, health and fitness outcomes in sedentary and underactive older adults aged 55–65. *Patient Educ. Couns*. **2006**, *62*, 132–141.
- De Nooijer, J.; de Vet, E.; Brug, J.; de Vries, N.K. Do implementation intentions help to turn good intentions into higher fruit intakes? *J. Nutr. Educ. Behav*. **2006**, *38*, 25–29.
- Delichatsios, H.K.; Friedman, R.H.; Glanz, K.; Tennstedt, S.; Smigelski, C.; Pinto, B.M.; Kelley, H.; Gillman, M.W. Randomized trial of a "talking computer" to improve adults' eating habits. *Amer. J. Health Promot*. **2001**, *15*, 215–224.
- Delichatsios, H.K.; Hunt, M.K.; Lobb, R.; Emmons, K.; Gillman, M.W. EatSmart: Efficacy of a multifaceted preventive nutrition intervention in clinical practice. *Prev. Med.* **2001**, *33*, 91–98.
- Duru, O.K.; Sarkisian, C.A.; Leng, M.; Mangione, C.M. Sisters in motion: A randomized controlled trial of a faith-based physical activity intervention. *J. Amer. Geriatr. Soc*. **2010**, *58*, 1863–1869.
- Dzator, J.A.; Hendrie, D.; Burke, V.; Gianguilio, N.; Gillam, H.F.; Beilin, L.J.; Houghton, S. A randomized trial of interactive group sessions achieved greater improvements in nutrition and physical activity at a tiny increase in cost. *J. Clin. Epidemiol*. **2004**, *57*, 610–619.
- Eaton, C.B.; Lapane, K.L.; Garber, C.E.; Gans, K.M.; Lasater, T.M.; Carleton, R.A. Effects of a community-based intervention on physical activity: The pawtucket heart health program. *Amer. J. Public Health* **1999**, *89*, 1741–1744.
- Elder, J.P.; Ayala, G.X.; Campbell, N.R.; Slymen, D.; Lopez-Madurga, E.T.; Engelberg, M.; Baquero, B. Interpersonal and print nutrition communication for a Spanish-dominant Latino population: Secretos de la Buena Vida. *Health Psychol*. **2005**, *24*, 49–57.
- Estabrooks, P.A.; Bradshaw, M.; Dzewaltowski, D.A.; Smith-Ray, R.L. Determining the impact of walk Kansas: Applying a team-building approach to community physical activity promotion. *Ann. Behav. Med.* **2008**, *36*, 1–12.
- Fahrenwald, N.L.; Atwood, J.R.; Walker, S.N.; Johnson, D.R.; Berg, K. A randomized pilot test of "Moms on the Move": A physical activity intervention for WIC mothers. *Ann. Behav. Med*. **2004**, *27*, 82–90.
- Farley, T.A.; Meriwether, R.A.; Baker, E.T.; Watkins, L.T.; Johnson, C.C.; Webber, L.S. Safe play spaces to promote physical activity in inner-city children: Results from a pilot study of an environmental intervention. *Amer. J. Public Health* **2007**, *97*, 1625–1631.
- Ferney, S.L.; Marshall, A.L.; Eakin, E.G.; Owen, N. Randomized trial of a neighborhood environment-focused physical activity website intervention. *Prev. Med.* **2009**, *48*, 144–150.
- Fisher, K.J.; Li, F. A community-based walking trial to improve neighborhood quality of life in older adults: A multilevel analysis. *Ann. Behav. Med.* **2004**, *28*, 186–194.

- Fjeldsoe, B.S.; Miller, Y.D.; Marshall, A.L. MobileMums: A randomized controlled trial of an SMS-based physical activity intervention. *Ann. Behav. Med.* **2010**, *39*, 101–111.
- Folta, S.C.; Lichtenstein, A.H.; Seguin, R.A.; Goldberg, J.P.; Kuder, J.F.; Nelson, M.E. The strongwomen-healthy hearts program: Reducing cardiovascular disease risk factors in rural sedentary, overweight, and obese midlife and older women. *Amer. J. Public Health* **2009**, *99*, 1271–1277.
- French, S.A.; Gerlach, A.F.; Mitchell, N.R.; Hannan, P.J.; Welsh, E.M. Household obesity prevention: Take action—A group-randomized trial. *Obesity* **2011**, *19*, 2082–2088.
- French, S.A.; Story, M.; Fulkerson, J.A.; Himes, J.H.; Hannan, P.; Neumark-Sztainer, D.; Ensrud, K. Increasing weight-bearing physical activity and calcium-rich foods to promote bone mass gains among 9–11 year old girls: Outcomes of the cal-girls study. *Int. J. Behav. Nutr. Phys. Act.* **2005**, *2*, doi:10.1186/1479-5868-2-8.
- Fu, D.; Fu, H.; McGowan, P.; Shen, Y.E.; Zhu, L.; Yang, H.; Mao, J.; Zhu, S.; Ding, Y.; Wei, Z. Implementation and quantitative evaluation of chronic disease self-management programme in Shanghai, China: Randomized controlled trial. *Bull. WHO* **2003**, *81*, 174–182.
- Gentile, D.A.; Welk, G.; Eisenmann, J.C.; Reimer, R.A.; Walsh, D.A.; Russell, D.W.; Callahan, R.; Walsh, M.; Strickland, S.; Fritz, K. Evaluation of a multiple ecological level child obesity prevention program: Switch what you do, view, and chew. *BMC Med.* **2009**, *7*, doi:10.1186/1741-7015-7-49.
- Goodman, R.M.; Wheeler, F.C.; Lee, P.R. Evaluation of the heart to heart project: Lessons from a community-based chronic disease prevention project. *Amer. J. Health Promot.* **1995**, *9*, 443–455.
- Greaney, M.L.; Riebe, D.; Ewing Garber, C.; Rossi, J.S.; Lees, F.D.; Burbank, P.A.; Nigg, C.R.; Ferrone, C.L.; Clark, P.G. Long-term effects of a stage-based intervention for changing exercise intentions and behavior in older adults. *Gerontologist* **2008**, *48*, 358–367.
- Gu, W. Study on the risk factors of hypertension and the evaluation of the comprehensive intervention in the rural community of Jiaxing. Master Thesis of School of Public Health, Zhejiang University, Zhejiang, China, 2006.
- Guo, Y.; Wang, H.; Yan, H.; Zhang, F.; Gao, C.; Xiang, N.; Fu, X.; Han, F. Evaluation on the effect of hypertension health education in country community. *Chin. J. Health Educ.* **2006**, *22*, 770–772.
- Haire-Joshu, D.; Nanney, M.S.; Elliott, M.; Davey, C.; Caito, N.; Loman, D.; Brownson, R.C.; Kreuter, M.W. The use of mentoring programs to improve energy balance behaviors in high-risk children. *Obesity* **2010**, *18*, S75–S83.
- Havas, S.; Anliker, J.; Damron, D.; Langenberg, P.; Ballesteros, M.; Feldman, R. Final results of the maryland wic 5-a-day promotion program. *Amer. J. Public Health* **1998**, *88*, 1161–1167.
- Hivert, M.F.; Langlois, M.F.; Berard, P.; Cuerrier, J.P.; Carpentier, A.C. Prevention of weight gain in young adults through a seminar-based intervention program. *Int. J. Obesity* **2007**, *31*, 1262–1269.
- Hogue, P.A. The Effects of Buddy Support on Physical Activity in African American Women. PhD Thesis, University of Toledo, Toledo, OH, USA, August 2007.

- Hovell, M.F.; Mulvihill, M.M.; Buono, M.J.; Liles, S.; Schade, D.H.; Washington, T.A.; Manzano, R.; Sallis, J.F. Culturally tailored aerobic exercise intervention for low-income Latinas. *Amer. J. Health Promot.* **2008**, *22*, 155–163.
- Hsu, H.; Wang, C.-H., Chen, Y.-C.; Chang, M.; Wang, J. Evaluation of a community-based aging intervention program. *Educ. Gerontol.* **2010**, *36*, 547–572.
- Hurling, R.; Catt, M.; Boni, M.D.; Fairley, B.W.; Hurst, T.; Murray, P.; Richardson, A.; Sodhi, J.S. Using internet and mobile phone technology to deliver an automated physical activity program: Randomized controlled trial. *J. Med. Internet Res.* **2007**, *9*, doi:10.2196/jmir.9.2.e7.
- Inoue, S.; Odagiri, Y.; Wakui, S.; Katoh, R.; Moriguchi, T.; Ohya, Y.; Shimomitsu, T. Randomized controlled trial to evaluate the effect of a physical activity intervention program based on behavioural medicine. *J. Tokyo Med.* **2003**, *61*, 154–65.
- Insull, W., Jr.; Henderson, M.M.; Prentice, R.L.; Thompson, D.J.; Clifford, C.; Goldman, S.; Gorbach, S.; Moskowitz, M.; Thompson, R.; Woods, M. Results of a randomized feasibility study of a low-fat diet. *Arch. Intern. Med.* **1990**, *150*, 421–427.
- Jacobs, A.D.; Ammerman, A.S.; Ennett, S.T.; Campbell, M.K.; Tawney, K.W.; Aytur, S.A.; Marshall, S.W.; Will, J.C.; Rosamond, W.D. Effects of a tailored follow-up intervention on health behaviors, beliefs, and attitudes. *J. Womens Health* **2004**, *13*, 557–568.
- Jago, R.; Baranowski, T.; Baranowski, J.C.; Thompson, D.; Cullen, K.W.; Watson, K.; Liu, Y. Fit for life boy scout badge: Outcome evaluation of a troop and internet intervention. *Prev. Med.* **2006**, *42*, 181–187.
- Jancey, J.M.; Lee, A.H.; Howat, P.A.; Clarke, A.; Wang, K.; Shilton, T. The effectiveness of a physical activity intervention for seniors. *Amer. J. Health Promot.* **2008**, *22*, 318–321.
- Jarrett, R.J.; Keen, H.; Murrells, T. Changes in blood pressure and body weight over ten years in men selected for glucose intolerance. *J. Epidemiol. Community Health* **1987**, *41*, 145–151.
- Jenum, A.K.; Anderssen, S.A.; Birkeland, K.I.; Holme, I.; Graff-Iversen, S.; Lorentzen, C.; Ommundsen, Y.; Raastad, T.; Odegaard, A.K.; Bahr, R. Promoting physical activity in a low-income multiethnic district: Effects of a community intervention study to reduce risk factors for type 2 diabetes and cardiovascular disease: A community intervention reducing inactivity. *Diabetes Care* **2006**, *29*, 1605–1612.
- Jiang, B.; Wang, W.; Wu, S. The effects of community intervention measures on prevention and control of hypertension. *Chin. J. Prev. Control Chronic Non-communicable Dis.* **2008**, *16*, 254–257.
- Jorna, M.; Ball, K.; Salmon, J. Effects of a holistic health program on women's physical activity and mental and spiritual health. *J. Sci. Med. Sport* **2006**, *9*, 395–401.
- Kellar, I.; Abraham, C. Randomized controlled trial of a brief research-based intervention promoting fruit and vegetable consumption. *Brit. J. Health. Psychol.* **2005**, *10*, 543–558.
- Kerr, J.; Eves, F.; Carroll, D. Can posters prompt stair use in a worksite environment ? *J. Occup. Health* **2001**, *43*, 205–207.
- Kerr, J.; Eves, F.; Carroll, D. Encouraging stair use: Stair-riser banners are better than posters. *Amer. J. Public Health* **2001**, *91*, 1192–1193.
- Kerr, J.; Eves, F.F.; Carroll, D. Getting more people on the stairs: The impact of a new message format. *J. Health. Psychol.* **2001**, *6*, 495–500.

- Kerr, J.; Eves, F.F.; Carroll, D. The influence of poster prompts on stair use: The effects of setting, poster size and content. *Brit. J. Health. Psychol.* **2001**, *6*, 397–405.
- King, A.C.; Ahn, D.K.; Oliveira, B.M.; Atienza, A.A.; Castro, C.M.; Gardner, C.D. Promoting physical activity through hand-held computer technology. *Amer. J. Prev. Med.* **2008**, *34*, 138–142.
- King, A.C.; Friedman, R.; Marcus, B.; Castro, C.; Napolitano, M.; Ahn, D.; Baker, L. Ongoing physical activity advice by humans versus computers: The community health advice by telephone (chat) trial. *Health Psychol.* **2007**, *26*, 718–727.
- Kinmonth, A.L.; Wareham, N.J.; Hardeman, W.; Sutton, S.; Prevost, A.T.; Fanshawe, T.; Williams, K.M.; Ekelund, U.; Spiegelhalter, D.; Griffin, S.J. Efficacy of a theory-based behavioural intervention to increase physical activity in an at-risk group in primary care (ProActive UK): A randomised trial. *Lancet* **2008**, *371*, 41–48.
- Kloek, G.C.; van Lenthe, F.J.; van Nierop, P.W.; Koelen, M.A.; Mackenbach, J.P. Impact evaluation of a Dutch community intervention to improve health-related behaviour in deprived neighbourhoods. *Health Place* **2006**, *12*, 665–677.
- Knowler, W.C.; Barrett-Connor, E.; Fowler, S.E.; Hamman, R.F.; Lachin, J.M.; Walker, E.A.; Nathan, D.M.; Diabetes Prevention Program Research Group. Reduction in the incidence of type 2 diabetes with lifestyle intervention or metformin. *N. Engl. J. Med.* **2002**, *346*, 393–403.
- Koizumi, D.; Rogers, N.L.; Rogers, M.E.; Islam, M.M.; Kusunoki, M.; Takeshima, N. Efficacy of an accelerometer-guided physical activity intervention in community-dwelling older women. *J. Phys. Act. Health* **2009**, *6*, 467–474.
- Krieger, J.; Rabkin, J.; Sharify, D.; Song, L. High point walking for health: creating built and social environments that support walking in a public housing community. *Amer. J. Public Health* **2009**, *99*, S593–S599.
- Kriska, A.M.; Bayles, C.; Cauley, J.A.; LaPorte, R.E.; Sandler, R.B.; Pambianco, G. A randomized exercise trial in older women: increased activity over two years and the factors associated with compliance. *Med. Sci. Sport. Exercise* **1986**, *18*, 557–562.
- Kroeze, W.; Oenema, A.; Campbell, M.; Brug, J. The efficacy of web-based and print-delivered computer-tailored interventions to reduce fat intake: Results of a randomized, controlled trial. *J. Nutr. Educ. Behav.* **2008**, *40*, 226–236.
- Kumpusalo, E.; Neittaanmaki, L.; Halonen, P.; Pekkarinen, H. Finnish Healthy Village Study: Impact and outcomes of a low-cost local health promotion programme. *Health Promot. International* **1996**, *11*, 105–115.
- Lamb, S.E.; Bartlett, H.P.; Ashley, A.; Bird, W. Can lay-led walking programmes increase physical activity in middle aged adults? A randomised controlled trial. *J. Epidemiol. Community Health* **2002**, *56*, 246–252.
- Lane, A.; Murphy, N.; Bauman, A.; Chey, T. Randomized controlled trial to increase physical activity among insufficiently active women following their participation in a mass event. *Health Educ. J.* **2010**, *69*, 287–296.

- Lee, R.E.; O'Connor, D.P.; Smith-Ray, R.; Mama, S.K.; Medina, A.V.; Reese-Smith, J.Y.; Banda, J.A.; Layne, C.S.; Brosnan, M.; Cubbin, C.; *et al.* Mediating effects of group cohesion on physical activity and diet in women of color: Health is power. *Amer. J. Health Promot.* **2012**, *26*, 116–125.
- Levy, S.S.; Cardinal, B.J. Effects of a Self-determination theory-based mail-mediated intervention on adults' exercise behavior. *Amer. J. Health Promot.* **2004**, *18*, 345–349.
- Lindahl, B.; Nilsson, T.K.; Jansson, J.-H.; Asplund, K.; Hallmans, G. Improved fibrinolysis by intense lifestyle intervention. A randomized trial in subjects with impaired glucose tolerance. *J. Intern. Med.* **1999**, *246*, 105–112.
- Lombard, C.; Deeks, A.; Jolley, D.; Ball, K.; Teede, H. A low intensity, community based lifestyle programme to prevent weight gain in women with young children: Cluster randomised controlled trial. *BMJ* **2010**, *341*, doi:10.1136/bmj.c3215.
- Lowther, M.; Mutrie, N.; Scott, E.M. Promoting physical activity in a socially and economically deprived community: A 12 month randomized control trial of fitness assessment and exercise consultation. *J. Sport. Sci.* **2002**, *20*, 577–588.
- Lucumi, D.I.; Sarmiento, O.L.; Forero, R.; Gomez, L.F.; Espinosa, G. Community intervention to promote consumption of fruits and vegetables, smoke-free homes, and physical activity among home caregivers in Bogota, Colombia. *Prev. Chronic Dis.* **2006**, *3*, 1–13.
- Luepker, R.V.; Murray, D.M.; Jacobs, D.R.,Jr.; Mittelmark, M.B.; Bracht, N.; Carlaw, R.; Crow, R.; Elmer, P.; Finnegan, J.; Folsom, A.R. Community education for cardiovascular disease prevention: Risk factor changes in the minnesota heart health program. *Amer. J. Public Health* **1994**, *84*, 1383–1393.
- Lupton, B.S.; Fonnebo, V.; Sogaard, A.J.; Finnmark Intervention Study. The finnmark intervention study: Is it possible to change cvd risk factors by community-based intervention in an arctic village in crisis? *Scand. J. Public Health* **2003**, *31*, 178–186.
- Lupton, B.S.; Fonnebo, V.; Sogaard, A.J.; Langfeldt, E. The finnmark intervention study. Better health for the fishery population in an arctic village in North Norway. *Scand. J. Prim. Health Care* **2002**, *20*, 213–218.
- Macias-Cervantes, M.H.; Malacara, J.M.; Garay-Sevilla, M.E.; Diaz-Cisneros, F.J. Effect of recreational physical activity on insulin levels in Mexican/Hispanic children. *Eur. J. Pediatr.* **2009**, *168*, 1195–1202.
- Maki, Y.; Ura, C.; Yamaguchi, T.; Murai, T.; Isahai, M.; Kaiho, A.; Yamagami, T.; Tanaka, S.; Miyamae, F.; Sugiyama, M.; *et al.* Effects of intervention using a community-based walking program for prevention of mental decline: A randomized controlled trial. *J. Amer. Geriatr. Soc.* **2012**, *60*, 505–510.
- Marcus, B.H.; Napolitano, M.A.; King, A.C.; Lewis, B.A.; Whiteley, J.A.; Albrecht, A.; Parisi, A.; Bock, B.; Pinto, B.; Sciamanna, C.; *et al.* Telephone *vs.* print delivery of an individualized motivationally tailored physical activity intervention: Project STRIDE. *Health Psychol.* **2007**, *26*, 401–409.
- Marshall, A.L.; Bauman, A.E.; Owen, N.; Booth, M.L.; Crawford, D.; Marcus, B.H. Reaching out to promote physical activity in Australia: A statewide randomized controlled trial of a stage-targeted intervention. *Amer. J. Health Promot.* **2004**, *18*, 283–287.

- Marshall, A.L.; Bauman, A.E.; Patch, C.; Wilson, J.; Chen, J. Can motivational signs prompt increases in incidental physical activity in an australian health-care facility? *Health Educ. Res.* **2002**, *17*, 743–749.
- Marshall, A.L.; Leslie, E.R.; Bauman, A.E.; Marcus, B.H.; Owen, N. Print *vs*. website physical activity programs: A randomized trial. *Amer. J. Prev. Med*. **2003**, *25*, 88–94.
- Martinson, B.C.; Crain, A.L.; Sherwood, N.E.; Hayes, M.; Pronk, N.P.; O'Connor, P.J. Maintaining physical activity among older adults: Six-month outcomes of the keep active Minnesota randomized controlled trial. *Prev. Med*. **2008**, *46*, 111–119.
- Mayer, J.A.; Jermanovich, A.; Wright, B.L.; Elder, J.P.; Drew, J.A.; Williams, S.J. Changes in health behaviors of older adults: The San Diego medicare preventive health project. *Prev. Med.* **1994**, *23*, 127–133.
- McAuley, E.; Courneya, K.S.; Rudolph, D.L.; Lox, C.L. Enhancing exercise adherence in middle-aged males and females. *Prev. Med*. **1994**, *23*, 498–506.
- Mensink, M.; Blaak, E.E.; Corpeleijn, E.; Saris, W.H.; de Bruin, T.W.; Feskens, E.J. Lifestyle intervention according to general recommendations improves glucose tolerance. *Obes. Res.* **2003**, *11*, 1588–1596.
- Merom, D.; Rissel, C.; Phongsavan, P.; Smith, B.J.; van Kemenade, C.; Brown, W.J.; Bauman, A.E. Promoting walking with pedometers in the community: The step-by-step trial. *Amer. J. Prev. Med*. **2007**, *32*, 290–297.
- Miller, Y.D.; Trost, S.G.; Brown, W.J. Mediators of physical activity behavior change among women with young children. *Amer. J. Prev. Med*. **2002**, *23*, 98–103.
- Nafziger, A.N.; Erb, T.A.; Jenkins, P.L.; Lewis, C.; Pearson, T.A. The otsego-schoharie healthy heart program: Prevention of cardiovascular disease in the rural USA. *Scand. J. Public. Health. Suppl.* **2001**, *56*, 21–32.
- Newton, R.L., Jr.; Perri, M.G. A randomized pilot trial of exercise promotion in sedentary African-American adults. *Ethn. Dis*. **2004**, *14*, 548–557.
- Nguyên, M.; Gauvin, L.; Martineau, I.; Grignon, R. Promoting physical activity at the community level: Insights into health promotion practice from the laval walking clubs experience. *Health Promot. Pract*. **2002**, *3*, 485–496.
- Nies, M.A.; Chruscial, H.L.; Hepworth, J.T. An intervention to promote walking in sedentary women in the community. *Amer. J. Health Behav*. **2003**, *27*, 524–535.
- Nies, M.A.; Motyka, C.L. Factors contributing to women's ability to maintain a walking program. *J. Holist. Nurs*. **2006**, *24*, 7–14.
- Nishtar, S.; Badar, A.; Kamal, M.U.; Iqbal, A.; Bajwa, R.; Shah, T.; Larik, Z.; Karim, F.; Mehmood, M.; Jehangir, H.; *et al.* The heartfile lodhran CVD prevention project—End of project evaluation. *Promot. Educ*. **2007**, *14*, 17–27.
- NSW Health Department. Walk it: Active Local Parks' Summary Report. **April 2002:36.**
- O'Loughlin, J.L.; Paradis, G.; Gray-Donald, K.; Renaud, L. The impact of a community-based heart disease prevention program in a low-income, inner-city neighbourhood. *Amer. J. Public Health* **1999**, *89*, 1819–1826.

- Olvera, N.; Bush, J.A.; Sharma, S.V.; Knox, B.B.; Scherer, R.L.; Butte, N.F. BOUNCE: A community-based mother-daughter healthy lifestyle intervention for low-income Latino families. Obesity **2010**, *18*, S102–S104.
- Opdenacker, J.; Boen, F.; Vanden Auweele, Y.; de Bourdeaudhuij, I. Effectiveness of a lifestyle physical activity intervention in a women's organization. *J. Womens Health* **2008**, *17*, 413–421.
- Osler, M.; Jespersen, N.B. The effect of a community-based cardiovascular disease prevention project in a Danish municipality. *Dan. Med. Bull.* **1993**, *40*, 485–489.
- Paineau, D.L.; Beaufils, F.; Boulier, A.; Cassuto, D.A.; Chwalow, J.; Combris, P.; Couet, C.; Jouret, B.; Lafay, L.; Laville, M.; *et al.* Family dietary coaching to improve nutritional intakes and body weight control: A randomized controlled trial. *Arch. Pediatr. Adolesc. Med.* **2008**, *162*, 34–43.
- Pan, X.R.; Li, G.W.; Hu, Y.H.; Wang, J.X.; Yang, W.Y.; An, Z.X.; Hu, Z.X.; Lin, J.; Xiao, J.Z.; Cao, H.B.; *et al.* Effects of diet and exercise in preventing NIDDM in people with impaired glucose tolerance. The Da Qing IGT and diabetes study. *Diabetes Care* **1997**, *20*, 537–544.
- Peterson, J.A.; Yates, B.C.; Atwood, J.R.; Hertzog, M. Effects of a physical activity intervention for women. *West. J. Nurs. Res.* **2005**, *27*, 93–110.
- Poston, W.S.; Haddock, C.K.; Olvera, N.E.; Suminski, R.R.; Reeves, R.S.; Dunn, J.K.; Hanis, C.L.; Foreyt, J.P. Evaluation of a culturally appropriate intervention to increase physical activity. *Amer. J. Health Behav.* **2001**, *25*, 396–406.
- Ramachandran, A.; Snehalatha, C.; Mary, S.; Mukesh, B.; Bhaskar, A.D.; Vijay, V.; Indian Diabetes Prevention Programme (IDPP). The Indian diabetes prevention programme shows that lifestyle modification and metformin prevent type 2 diabetes in Asian Indian subjects with impaired glucose tolerance (IDPP-1). *Diabetologia* **2006**, *49*, 289–297.
- Reger, B.; Cooper, L.; Booth-Butterfield, S.; Smith, H.; Bauman, A.; Wootan, M.; Middlestadt, S.; Marcus, B.; Greer, F. Wheeling walks: A community campaign using paid media to encourage walking among sedentary older adults. *Prev. Med.* **2002**, *35*, 285–292.
- Reger-Nash, B.; Bauman, A.; Booth-Butterfield, S.; Cooper, L.; Smith, H.; Chey, T.; Simon, K.J. Wheeling walks: Evaluation of a media-based community intervention. *Family Community Health* **2005**, *28*, 64–78.
- Reger-Nash, B.; Cooper, L.; Orren, J.; Cook, D. Marketing used to promote walking in McDowell county. *W.V. Med. J.* **2005**, *101*, 106–106.
- Resnick, B. Testing the effect of the WALC intervention on exercise adherence in older adults. J. *Gerontol. Nurs*. **2002**, *28*, 40–49.
- Resnick, B.; Luisi, D.; Vogel, A. Testing the senior exercise self-efficacy project (SESEP) for use with urban dwelling minority older adults. *Public Health Nurs*. **2008**, *25*, 221–234.
- Resnicow, K.; Jackson, A.; Blissett, D.; Wang, T.; McCarty, F.; Rahotep, S.; Periasamy, S. Results of the healthy body healthy spirit trial. *Health Psychol*. **2005**, *24*, 339–348.
- Resnicow, K.; Jackson, A.; Wang, T.; De, A.K.; McCarty, F.; Dudley, W.N.; Baranowski, T. A motivational interviewing intervention to increase fruit and vegetable intake through black churches: Results of the eat for life trial. *Amer. J. Public Health* **2001**, *91*, 1686–1693.

- Robinson, T.N.; Killen, J.D.; Kraemer, H.C.; Wilson, D.M.; Matheson, D.M.; Haskell, W.L.; Pruitt, L.A.; Powell, T.M.; Owens, A.S.; Thompson, N.S.; *et al.* Dance and reducing television viewing to prevent weight gain in african-american girls: The Stanford GEMS pilot study. *Ethn. Dis*. **2003**, *13*, S65–S77.
- Rodearmel, S.J.; Wyatt, H.R.; Barry, M.J.; Dong, F.; Pan, D.; Israel, R.G.; Cho, S.S.; McBurney, M.I.; Hill, J.O. A family-based approach to preventing excessive weight gain. *Obesity* **2006**, *14*, 1392–1401.
- Rogers, T.M. Effectiveness of a Walking Club and a Self-Directed Physical Activity Program in Increasing Moderate Intensity Physical Activity among African-American Females. PhD Thesis, University of Oregon, Eugene, OR, USA, 1997.
- Rosenkranz, R.R.; Behrens, T.K.; Dzewaltowski, D.A. A group-randomized controlled trial for health promotion in girl scouts: Healthier troops in a SNAP (Scouting Nutrition & Activity Program). *BMC Public Health* **2010**, *10*, doi:10.1186/1471-2458-10-81.
- Rovniak, L.S.; Hovell, M.F.; Wojcik, J.R.; Winett, R.A.; Martinez-Donate, A.P. Enhancing Theoretical Fidelity: An e-mail-based walking program demonstration. *Amer. J. Health Promot.* **2005**, *20*, 85–95.
- Russell, W.D.; Dzewaltowski, D.A.; Ryan, G.J. The effectiveness of a point-of-decision prompt in deterring sedentary behavior. *Amer. J. Health Promot*. **1999**, *13*, 257–259.
- Russell, W.D.; Hutchinson, J. Comparison of health promotion and deterrent prompts in increasing use of stairs over escalators. *Percept. Mot. Skills* **2000**, *91*, 55–61.
- Sanigorski, A.M.; Bell, A.C.; Kremer, P.J.; Cuttler, R.; Swinburn, B.A. Reducing unhealthy weight gain in children through community capacity-building: Results of a quasi-experimental intervention program, be active eat well. *Int. J. Obes*. **2008**, *32*, 1060–1067.
- Sarrafzadegan, N.; Kelishadi, R.; Esmaillzadeh, A.; Mohammadifard, N.; Rabiei, K.; Roohafza, H.; Azadbakht, L.; Bahonar, A.; Sadri, G.; Amani, A.; *et al.* Do lifestyle interventions work in developing countries? Findings from the Isfahan healthy heart program in the Islamic Republic of Iran. *Bull. WHO* **2009**, *87*, 39–50.
- Schneider, J.K.; Mercer, G.T.; Herning, M.; Smith, C.A.; Prysak, M.D. Promoting exercise behavior in older adults: Using a cognitive behavioral intervention. *J. Gerontol. Nurs*. **2004**, *30*, 45–53.
- Sevick, M.A.; Napolitano, M.A.; Papandonatos, G.D.; Gordon, A.J.; Reiser, L.M.; Marcus, B.H. Cost-effectiveness of alternative approaches for motivating activity in sedentary adults: Results of project STRIDE. *Prev. Med*. **2007**, *45*, 54–61.
- Shirazi, K.K.; Wallace, L.M.; Niknami, S.; Hidarnia, A.; Torkaman, G.; Gilchrist, M.; Faghihzadeh, S. A home-based, transtheoretical change model designed strength training intervention to increase exercise to prevent osteoporosis in iranian women aged 40–65 years: A randomized controlled trial. *Health Educ. Res*. **2007**, *22*, 305–317.
- Sidman, C.L.; Corbin, C.B.; Le Masurier, G. Promoting physical activity among sedentary women using pedometers. *Res. Quart. Exercise Sport* **2004**, *75*, 122–129.
- Simmons, D.; Voyle, J.A.; Fou, F.; Feo, S.; Leakehe, L. Tale of two churches: Differential impact of a church-based diabetes control programme among Pacific islands people in New Zealand. *Diabet. Med*. **2004**, *21*, 122–128.

- Simon, C.; Schweitzer, B.; Oujaa, M.; Wagner, A.; Arveiler, D.; Triby, E.; Copin, N.; Blanc, S.; Platat, C. Successful overweight prevention in adolescents by increasing physical activity: A 4-year randomized controlled intervention. *Int. J. Obesity* **2008**, *32*, 1489–1498.
- Speck, B.J.; Hines-Martin, V.; Stetson, B.A.; Looney, S.W. An environmental intervention aimed at increasing physical activity levels in low-income women. *J. Cardiovasc. Nurs*. **2007**, *22*, 263–271.
- Speck, B.J.; Looney, S.W. Effects of a minimal intervention to increase physical activity in women: Daily activity records. *Nurs. Res.* **2001**, *50*, 374–378.
- Spittaels, H.; De Bourdeaudhuij, I.; Brug, J.; Vandelanotte, C. Effectiveness of an online computer-tailored physical activity intervention in a real-life setting. *Health Educ. Res*. **2007**, *22*, 385–396.
- Staten, L.K.; Scheu, L.L.; Bronson, D.; Pena, V.; Elenes, J. Pasos Adelante: The effectiveness of a community-based chronic disease prevention program. *Prev. Chronic Dis*. **2005**, *2*, 1–11.
- Steele, R.; Mummery, W.K.; Dwyer, T. Using the internet to promote physical activity: A randomized trial of intervention delivery modes. *J. Phys. Act. Health* **2007**, *4*, 245–260.
- Stevens, V.J.; Glasgow, R.E.; Toobert, D.J.; Karanja, N.; Smith, K.S. Randomized trial of a brief dietary intervention to decrease consumption of fat and increase consumption of fruits and vegetables. *Amer. J. Health Promot*. **2002**, *16*, 129–134.
- Stewart, A.L.; Mills, K.M.; Sepsis, P.G.; King, A.C.; McLellan, B.Y.; Roitz, K.; Ritter, P.L. Evaluation of CHAMPS, a physical activity promotion program for older adults. *Ann. Behav. Med.* **1997**, *19*, 353–361.
- Stoddard, A.M.; Palombo, R.; Troped, P.J.; Sorensen, G.; Will, J.C. Cardiovascular disease risk reduction: The Massachusetts WISEWOMAN project. *J. Womens Health* **2004**, *13*, 539–546.
- Stolley, M.R.; Fitzgibbon, M.L. Effects of an obesity prevention program on the eating behavior of African American mothers and daughters. *Health Educ. Behav*. **1997**, *24*, 152–164.
- Story, M.; Sherwood, N.E.; Himes, J.H.; Davis, M.; Jacobs, D.R.,Jr; Cartwright, Y.; Smyth, M.; Rochon, J. An after-school obesity prevention program for African-American girls: The Minnesota GEMS pilot study. *Ethn. Dis*. **2003***, 13*, S54–S64.
- Sullivan, T.; Allegrante, J.P.; Peterson, M.G.; Kovar, P.A.; MacKenzie, C.R. One-year followup of patients with osteoarthritis of the knee who participated in a program of supervised fitness walking and supportive patient education. *Arthritis Care Res*. **1998**, *11*, 228–233.
- Takeda, N.; Koichiro, O.; Sakai, K.; Itakura, M; Nakamura, Y. The effects of a group-based walking program on daily physical activity in middle-aged and older adults. *Int. J. Sport Health Sci*. **2011**, *9*, 39–48.
- Tan, E.J.; Xue, Q.L.; Li, T.; Carlson, M.C.; Fried, L.P. Volunteering: A physical activity intervention for older adults—The experience corps program in Baltimore. *J. Urban Health* **2006**, *83*, 954–969.
- Tanner, A.; Duhe, S.; Evans, A.; Condrasky, M. Using student-produced media to promote healthy eating. A pilot study on the effects of a media and nutrition intervention. *Sci. Commun*. **2008**, *30*, 108–125.

- Tate, D.F.; Jackvony, E.H.; Wing, R.R. A randomized trial comparing human e-mail counseling, computer-automated tailored counseling, and no counseling in an internet weight loss program. *Arch. Intern. Med.* **2006**, *166*, 1620–1625.
- Tate, D.F.; Wing, R.R.; Winett, R.A. Using internet technology to deliver a behavioral weight loss program. *JAMA* **2001**, *285*, 1172–1177.
- Taylor, R.W.; McAuley, K.A.; Barbezat, W.; Strong, A.; Williams, S.M.; Mann, J.I. APPLE Project: 2-year findings of a community-based obesity prevention program in primary school age children. *Amer. J. Clin. Nutr.* **2007**, *86*, 735–742.
- Teri, L.; McCurry, S.M.; Logsdon, R.G.; Gibbons, L.E.; Buchner, D.M.; Larson, E.B. A randomized controlled clinical trial of the seattle protocol for activity in older adults. *J. Amer. Geriatr. Soc.* **2011**, *59*, 1188–1196.
- Thomas, G.N.; Macfarlane, D.J.; Guo, B.; Cheung, B.M.; McGhee, S.M.; Chou, K.L.; Deeks, J.J.; Lam, T.H.; Tomlinson, B. Health promotion in older Chinese: A 12-month cluster randomized controlled trial of pedometry and "peer support". *Med. Sci. Sport. Exercise* **2012**, *44*, 1157–1166.
- Thompson, J.L.; Allen, P.; Helitzer, D.L.; Qualls, C.; Whyte, A.N.; Wolfe, V.K.; Herman, C.J. Reducing diabetes risk in american indian women. *Amer. J. Prev. Med.* **2008**, *34*, 192–201.
- Tuomilehto, J.; Lindstrom, J.; Eriksson, J.G.; Valle, T.T.; Hamalainen, H.; Ilanne-Parikka, P.; Keinanen-Kiukaanniemi, S.; Laakso, M.; Louheranta, A.; Rastas, M.; *et al.* Prevention of type 2 diabetes mellitus by changes in lifestyle among subjects with impaired glucose tolerance. *N. Engl. J. Med.* **2001**, *344*, 1343–1350.
- Utter, J.; Scragg, R.; Robinson, E.; Warbrick, J.; Faeamani, G.; Foroughian, S.; Dewes, O.; Moodie, M.; Swinburn, B.A. Evaluation of the living 4 life project: A youth-led, school-based obesity prevention study. *Obes. Rev.* **2011**, *12*, S51–S60.
- Vandelanotte, C.; de Bourdeaudhuij, I.; Sallis, J.F.; Spittaels, H.; Brug, J. Efficacy of sequential or simultaneous interactive computer-tailored interventions for increasing physical activity and decreasing fat intake. *Ann. Behav. Med.* **2005**, *29*, 138–146.
- Walker, S.N.; Pullen, C.H.; Boeckner, L.; Hageman, P.A.; Hertzog, M.; Oberdorfer, M.K.; Rutledge, M.J. Clinical trial of tailored activity and eating newsletters with older rural women. *Nurs. Res.* **2009**, *58*, 74–85.
- Watson, N.; Milat, A.J.; Thomas, M.; Currie, J. The feasibility and effectiveness of pram walking groups for postpartum women in western Sydney. *Health Promot. J. Australia* **2005**, *16*, 93–99.
- Wendel-Vos, G.C.; Dutman, A.E.; Verschuren, W.M.; Ronckers, E.T.; Ament, A.; van Assema, P.; van Ree, J.; Ruland, E.C.; Schuit, A.J. Lifestyle factors of a five-year community-intervention program: The Hartslag Limburg intervention. *Amer. J. Prev. Med.* **2009**, *37*, 50–56.
- Wilbur, J.; McDevitt, J.H.; Wang, E.; Dancy, B.L.; Miller, A.M.; Briller, J.; Ingram, D.L.; Nicola, T.L.; Ju, S.; Lee, H. Outcomes of a home-based walking program for African-American women. *Amer. J. Health Promot.* **2008**, *22*, 307–317.
- Wilcox, S.; Laken, M.; Bopp, M.; Gethers, O.; Huang, P.; McClorin, L.; Parrott, A.W.; Swinton, R.; Yancey, A. Increasing physical activity among church members: Community-based participatory research. *Amer. J. Prev. Med.* **2007**, *32*, 131–138.

- Williams, B.; Bezner, J.; Chesbro, S.; Leavitt, R. The effect of a behavioral contract on adherence to a walking program in postmenopausal African American women. *Top. Geriatr. Rehabil.* **2005**, *21*, 332–342.
- Winett, R.A.; Anderson, E.S.; Wojcik, J.R.; Winett, S.G.; Bowden, T. Guide to health: Nutrition and physical activity outcomes of a group-randomized trial of an internet-based intervention in churches. *Ann. Behav. Med.* **2007**, *33*, 251–261.
- Wing, R.R.; Tate, D.F.; Gorin, A.A.; Raynor, H.A.; Fava, J.L. A self-regulation program for maintenance of weight loss. *N. Engl. J. Med.* **2006**, *355*, 1563–1571.
- Young, D.R.; Haskell, W.L.; Taylor, C.B.; Fortmann, S.P. Effect of community health education on physical activity knowledge, attitudes, and behavior. The Stanford five-city project. *Amer. J. Epidemiol.* **1996**, *144*, 264–274.
- Zhang, Y.; Zhao, Z.T.; Hao, F.R.; Jia, C.X.; Wang, S.M.; Feng, Y.Q. Effectiveness of diabetes mellitus community intervention on urban population's obesity and related factors. *Chin. J. Public Health* **2003**, *19*, 1396–1398.
- Zhang, Y.; Zhao, Z.T.; Li, G.R.; Hao, F.R.; Wang, S.M.; Pan, Y.Z.; Jia, C.X.; Feng, Y.Q. Effectiveness of community intervention on population's diabetes mellitus knowledge and its influencing factors. *Chin. J. Public Health* **2003**, *19*, 888–889.
- Zoellner, J.; Connell, C.; Powers, A.; Avis-Williams, A.; Yadrick, K.; Bogle, M.L. Does a Six-month pedometer intervention improve physical activity and health among vulnerable African Americans? A feasibility study. *J. Phys. Act. Health* **2010**, *7*, 224–231.

The reference list includes the systematic reviews and meta-analyses which were selected for the review of reviews.

References

1. Bleich, A.N.; Segal, J.; Wu, Y.; Wilson, R.; Wand, Y. Systematic review of community-based childhood obesity prevention studies. *Pediatrics* **2013**, *132*, 201–210.
2. Hendrie, G.A.; Brindal, E.; Corsini, N.; Gardner, C.; Baird, D.; Golley, R.K. Combined home and school obesity prevention interventions for children: What behavior change strategies and intervention characteristics are associated with effectiveness? *Health Educ. Behav.* **2012**, *39*, 159–171.
3. Kellou, N.; Sandalinas, F.; Copin, N.; Simon, C. Prevention of unhealthy weight in children by promoting physical activity using a socio-ecological approach: What can we learn from interventions studies? *Diabetes Metabol.* **2014**, *593*, doi:10.1016/j.diabet.2014.01.002.
4. Van Sluijs, E.M.; McMinn, A.M.; Griffin, S.J. Effectiveness of interventions to promote physical activity in children and adolescents: Systematic review of controlled trials. *BMJ* **2007**, *335*, doi:10.1136/bmj.39320.843947.BE.
5. Van Sluijs, E.M.; Kriemler, S.; McMinn, A.M. The effect of community and family interventions on young people's physical activity levels: A review of reviews and updated systematic review. *Brit. J. Sport. Med.* **2011**, *45*, 914–922.
6. Waters, E.; de Silva-Sanigorski, A.; Hall, B.J.; Brown, T.; Campbell, K.J.; Gao, Y.; Armstrong, R.; Prosser, L.; Summerbell, C.D. Interventions for preventing obesity in children. *Cochrane Database Syst. Rev.* **2011**, doi:10.1002/14651858.CD001871.pub3.

7. Wolfenden, L.; Wyse, R.; Nichols, M.; Allender, S.; Millar, L.; McElduff, P. A systematic review and meta-analysis of whole of community interventions to prevent excessive population weight gain. *Prev. Med.* **2014**, *62*, 193–200.
8. Michie, S.; Abraham, C.; Whittington, C.; McAteer, J.; Gupta, S. Effective techniques in healthy eating and physical activity interventions: A meta-regression. *Health Psychol.* **2009**, *28*, 690–701.
9. Kassavou, A.; Turner, A.; French, D.P. Do interventions to promote walking in groups increase physical activity? A meta-analysis. *Int. J. Behav. Nutr. Phys. Act.* **2013**, *10*, doi:10.1186/1479-5868-10-18.
10. Bock, C.; Jarczok, M.N.; Litaker, D. Community-based efforts to promote physical activity: A systematic review of interventions considering mode of delivery, study quality and population subgroups. *J. Sci. Med. Sport* **2014**, *17*, 276–282.
11. Webel, A.R.; Okonsky, J.; Trompeta, J.; Holzemer, W.L. A systematic review of the effectiveness of peer-based interventions on health-related behaviors in adults. *Amer. J. Public Health* **2010**, *100*, 247–253.
12. Soler, R.E.; Leeks, K.D.; Buchanan, L.R.; Brownson, R.C.; Heath, G.W.; Hopkins, D.H. Point-of-decision prompts to increase stair use: A systematic review update. *Amer. J. Prev. Med.* **2010,** *38*, S292–S300.
13. Baker, P.R.; Francis, D.P.; Soares, J.; Weightman, A.L.; Foster, C. Community wide interventions for increasing physical activity. *Cochrane Database Syst. Rev.* **2011**, doi:10.1002/14651858.CD008366.pub2.
14. Ogilvie, D.; Foster, C.E.; Rothnie, H.; Cavill, N.; Hamilton, V.; Fitzsimons, C.F.; Mutrie, N. Interventions to promote walking: Systematic review. *BMJ* **2007**, *334*, doi:10.1136/bmj.39198.722720.BE.
15. Garrett, S.; Elley, C.R.; Rose, S.B.; O'Dea, D.; Lawton, B.A.; Dowell, A.C. Are physical activity interventions in primary care and the community cost-effective? A systematic review of the evidence. *Brit. J. Gen. Pract.* **2011,** *61*, 125–133.
16. Cleland, V.; Granados, A; Crawford, D; Winzenberg, T.; Ball, K. Effectiveness of interventions to promote physical activity among socioeconomically disadvantaged women: A systematic review and meta-analysis. *Obes. Rev.* **2013**, *14*, 197–212.
17. Norris, S.L.; Zhang, X.; Avenell, A.; Gregg, E.; Schmid, C.H.; Lau, J. Long-term non-pharmacological weight loss interventions for adults with prediabetes. *Cochrane Database Syst. Rev.* **2009**, doi:10.1002/14651858.CD005270.
18. Baker, M.K.; Simpson, K.; Lloyd, B.; Bauman, A.E.; Singh, M.A. Behavioral strategies in diabetes prevention programs: A systematic review of randomized controlled trials. *Diabetes Res. Clin. Pract.* **2011**, *91*, 1–12.

© 2014 by the authors; licensee MDPI, Basel, Switzerland. This article is an open access article distributed under the terms and conditions of the Creative Commons Attribution license (http://creativecommons.org/licenses/by/3.0/).
